# Identification of Small RNAs in *Streptomyces clavuligerus* Using High-Resolution Transcriptomics and Expression Profiling During Clavulanic Acid Production

**DOI:** 10.3390/ijms252413472

**Published:** 2024-12-16

**Authors:** Carlos Caicedo-Montoya, Luisa F. Patiño, Rigoberto Ríos-Estepa

**Affiliations:** 1 Grupo de Bioprocesos, Departamento de Ingeniería Química, Universidad de Antioquia UdeA, Calle 70 No. 52-21, Medellín 050010, Colombia; candres.caicedo@udea.edu.co (C.C.-M.); luisa.patinoc@udea.edu.co (L.F.P.); 2 Grupo de Investigación en Simulación, Diseño, Control y Optimización de Procesos (SIDCOP), Departamento de Ingeniería Química, Universidad de Antioquia, Medellín 050010, Colombia

**Keywords:** *Streptomyces clavuligerus*, clavulanic acid, transcriptomics, small RNA, biosynthetic gene cluster

## Abstract

Small non-coding RNAs play a pivotal role in regulating various metabolic processes in both prokaryotic and eukaryotic organisms. However, knowledge about small RNAs (sRNAs) in *Streptomyces clavuligerus* (*S. clavuligerus*) is scarce. This study aimed to use cutting-edge bioinformatics tools and a compendium of RNA-seq data to predict the potential coding of sRNAs that might be present in the genome of *S. clavuligerus* ATCC 27064. In the genome of *S. clavuligerus*, 606 intergenic regions (IGRs) are conserved, and 272 possess a highly thermodynamically stable and conserved secondary structure, indicating the presence of non-coding RNA in these regions. The transcriptome assembly of *S. clavuligerus* showed that the genome is completely functional, as all the annotated genes are expressed under the conditions analyzed. From this assembly, transcripts originating from IGRs were labeled as putative sRNAs, and their differential expression during the growth curve of *S. clavuligerus* for clavulanic acid (CA) production was established. The interactome of these differentially expressed (DE) RNAs displayed the sRNAs as global regulators, as they can have multiple mRNA targets. The functional annotation of the target genes of DE sRNAs demonstrated that they are directly involved in secondary metabolite production. Specifically, two sRNA have the genes of the biosynthetic gene cluster of CA as targets. Thus, these molecules add an additional layer to the regulatory cascade for CA biosynthesis, and we propose them as targets for metabolic engineering to increase CA production.

## 1. Introduction

Transcription is pervasive in nature, meaning that most of the bacterial genome is transcribed into functional elements [1]. Historically, three main types of transcripts have been extensively studied due to their pivotal roles in gene expression and protein production: messenger RNA (mRNA), transfer RNA (tRNA), and ribosomal RNA (rRNA). Initially, the remaining parts of the genome were often considered “Junk DNA” because their functions were unknown. However, transcriptomic studies have revealed that many of these “non-coding” regions give rise to new molecules, drawing attention primarily due to the discovery of their regulatory functions. More accurately, these molecules are designated as non-coding RNAs (ncRNAs) [2]. Interestingly, while most of these RNAs do not encode proteins, there are notable exceptions. For instance, dual-function RNA regulators not only act as RNA regulators but also encode small proteins, such as SgrS in *E. coli*, RNAIII and Psm-mec in *Staphylococcus aureus*, Pel RNA in *Streptococcus pyogenes*, and SR1 in *Bacillus subtilis* [3,4].

Non-coding RNAs affect a huge variety of molecular processes, such as protein function, transcription initiation, mRNA stability, and translation initiation/elongation [5,6,7]. Although bacterial ncRNA-mediated regulatory mechanisms are expanding with the discovery of new ncRNAs [8], broadly, these regulators are currently classified according to their genomic context, that is, the site where they are encoded in the genome and their modes of actions.

First, there are ncRNAs that function by base-pairing with mRNA, resulting in the modulation of translation initiation and mRNA stability. In this group, we have trans-encoded small RNAs and cis-encoded regulatory RNAs. Cis-encoded regulatory RNAs originate from the opposite strand of a protein-coding gene. Hence, they are referred to as asRNAs and share full complementarity with their mRNA targets. In contrast, trans-encoded regulatory RNAs arise from intergenic regions (IGRs) of the genome and typically have partial sequence complementarity with their target mRNAs. In this scenario, they may have multiple targets scattered across different genomic loci [6,9,10,11]. Generally, cis-encoded RNAs exert negative regulatory effects on their targets, while trans-encoded, traditionally referred to as sRNAs, can have either positive or negative regulatory effects [12]. On the other hand, regarding their mode of action, ncRNAs can act in a trans or cis manner; thus, an asRNA can exhibit trans activity if it has targets located far away from the site where it is encoded [13]. In bacteria, ncRNAs span a wide range from 50 to 500 nucleotides, with notable examples as *Staphylococcus aureus* RNA III, which is 514 nucleotides long [14]. Finally, it is worth mentioning that other sources of bacterial ncRNAs have emerged as in the case of processed 3′ end fragments of mRNAs [15].

A second group, riboswitches, consists of RNA sequences located in the 5′ untranslated regions (UTRs) of mRNAs. Riboswitches regulate transcription, translation, or mRNA processing by adopting different conformational changes of the mRNA structure mediated by factors such as temperature or small molecules that bind to the riboswitch [12]. An essential characteristic of riboswitches is that RNA functions as both a sensor and an effector, demonstrating that a protein cofactor is not obligatory for regulation [16]. While riboswitches are not expressed as independent molecules, their structures and modes of action classify them as cis-encoded, cis-acting regulatory RNAs [13].

Another class of bacterial ncRNA comprises RNAs that bind to target proteins; some members of this group contribute essential functions to a ribonucleoprotein or act in a regulatory manner to antagonize the activities of their cognate proteins by mimicking the structures of other nucleic acids, e.g., the sRNAs CsrB and CsrC of *E. coli* influence the activity of CsrA, an RNA-binding protein that controls carbon usage and bacterial motility upon entering the stationary phase and other nutrient-limited conditions [17].

The fourth group of ncRNA regulators is associated with CRISPRs (clustered regularly interspaced short palindromic repeats). CRISPRs consist of an array of short, partially palindromic, repetitive non-coding DNA sequences interspersed by equally short variable sequences known as spacers. The latter are transcribed into CRISPR RNAs (crRNAs), which direct CRISPR-associated (Cas) nucleases to degrade the DNA or RNA of the invader, inhibiting the uptake of foreign DNA, thus preventing bacteriophage infection and plasmid conjugation [12,17,18].

The overall mechanism of translation repression by ncRNAs involves pairing directly to the ribosome-binding site (RBS) and competing with initiating ribosomes, an event that is often followed by rapid mRNA decay [19,20]. Moreover, several ncRNAs activate mRNA translation by pairing with the 5′-untranslated region (UTR) of their target mRNAs, disrupting inhibitory secondary structures [19].

The prediction of ncRNAs in bacteria can be achieved through bioinformatics methods. A genomic search of intergenic regions is the most-used strategy, though other features such as the presence of predicted Rho-independent terminators and promoters and genomic arrangements are also used to delimit the search [21]. To validate bioinformatics predictions, experimental procedures such as cloning ncRNA species from total RNA samples, a microarray analysis of intergenic regions, the immunoprecipitation of sRNAs associated with the RNA chaperone Hfq (necessary for the binding between sRNA and target mRNAs in most Gram-negative bacteria [22]), and a Northern blot analysis have been employed. Identifying and validating true ncRNAs has been challenging due to their varying sizes (between 50 to 500 nucleotides, as mentioned previously) and variable genomic locations in the 5′ and 3′ untranslated regions as well as intergenic regions [23]. The predominant alternative for studying ncRNAs in bacteria is the use of RNA-sequencing (RNA-seq) technology to identify expressed RNAs [24].

In the genus *Streptomyces*, which is the main source of antibiotics in the microbial world [25], many potential coding regions for ncRNAs have been identified [6,21,26,27,28]; the advent of the differential RNA-seq technique (dRNA-seq), which accurately predicts the boundaries of transcripts, has greatly contributed to this aim. However, the prediction of ncRNAs in Streptomycetes confronts several challenges: there is no Hfq orthologue in this genus, which is necessary for the binding between sRNA and target mRNAs in most Gram-negative bacteria [22]; the genomes are extremely GC rich, making the identification of rho-independent terminators not straightforward; the identification of promoter sequences is quite difficult because of the scarce characterization of these structures in the genus; and there is a strong expression dependency on the developmental stage at which samples are taken and the environmental conditions used in experiments [21,26,27,29]. For these reasons, a comprehensive annotation of sRNAs in *Streptomyces* remains limited.

A recent report [30] identified 27 novel regulatory RNAs in *S. clavuligerus*. However, despite this genome-wide determination, we strongly believe the genome of *S. clavuligerus* harbors additional ncRNAs, as the experimental conditions tested in the cited study are limited, including only four samples of *S. clavuligerus* at different time points during the growth curve. Furthermore, there are no reports about regulatory processes mediated by sRNA, so their function in the synthesis of clavulanic acid (CA) and other industrially important metabolites remains unknown.

**Figure 1:**
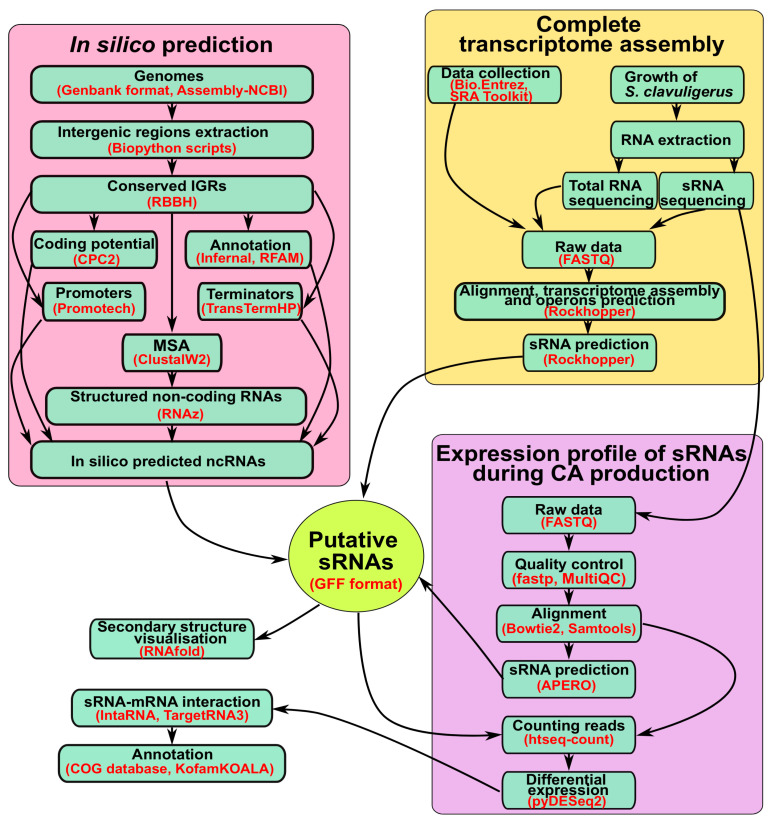
An overview of the workflow employed for the determination of sRNAs in *S. clavuligerus* and their putative role in CA production.

The aim of this study was to expand the search for sRNAs arising from intergenic regions in *S. clavuligerus* and to elucidate their influence on the production of secondary metabolites, considering CA as a case study. Deciphering the sRNAs and their interactome is an essential step towards understanding the role of sRNAs in cellular networks [31]. To achieve this, we performed a comparative genomic analysis of the IGRs in *S. clavuligerus* ATCC 27064, characterized for the presence of a 1.8-Mbp mega-plasmid (pCLA1) [32], against closely related strains and conducted a transcriptome assembly for identifying sRNAs unique in this strain. Finally, we established the differential expression of these annotated sRNAs to determine their relationship with CA production. Figure 1 shows a summary of the workflow employed in the present work.

## 2. Results

### 2.1. In Silico Prediction of Small RNAs

#### 2.1.1. General Features of Intergenic Regions in *Streptomyces clavuligerus*

The genome of *S. clavuligerus* ATCC 27064 contains 5819 intergenic regions (IGRs) ranging from 1 bp to 6243 bp, with a median length of 161 bp (Supplementary Figure S1A). These regions collectively amount to 1.354 MB, representing 15.85% of the complete genome. Additionally, we classified the IGRs as Double Promoter (DP), Double Terminator (DT), Co-oriented Forward (CO_F), and Co-oriented Reverse (CO_R) [33] based on the orientation of the genes flanking the IGRs (Supplementary Figure S1B,D). These classifications are similarly distributed across the genome of *S. clavuligerus*. The GC content of the IGRs does not differ significantly from the overall GC content of the genome, although some extreme values are observed, with a minimum of 42.9% and a maximum of 96.3% (Supplementary Figure S1C).

#### 2.1.2. Conserved IGRs in *S. clavuligerus* Are Enriched for sRNAs

We conducted a genome-wide comparison of the intergenic regions (IGRs) between *S. clavuligerus* ATCC 27064 and 36 closely related strains, determined from the alignment of 633 core genes and the subsequent construction of a high-confidence phylogenetic tree [34].

**Figure 2:**
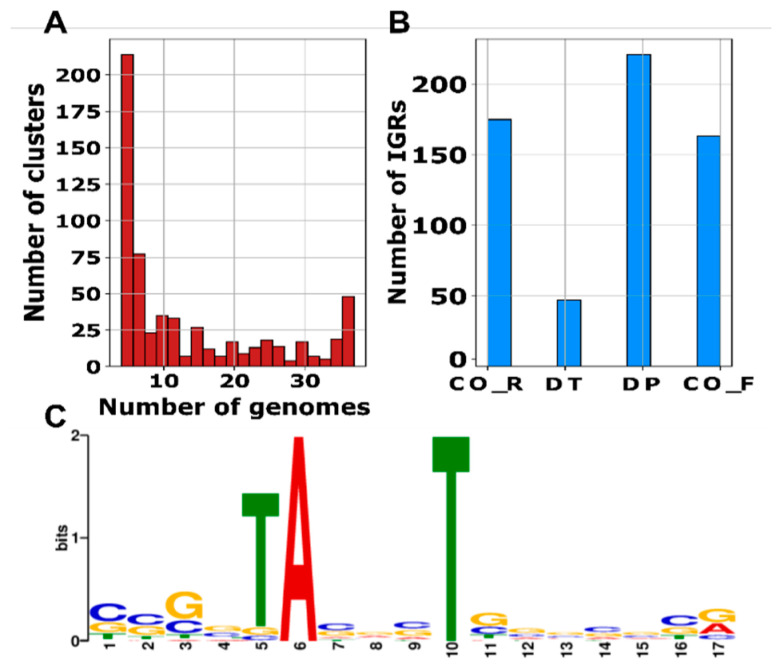
(**A**) The conservation of intergenic regions across the group of *Streptomyces* species considered in this study (Supplementary Table S1). The number of clusters represents the amount of IGR families or groups of IGRs detected. The number of genomes is the number of times an IGR appears in the studied genomes. (**B**) The classification of IGRs according to their neighboring genes for the conserved IGRs in *S. clavuligerus.* (**C**) Motif found from promoters detected in conserved IGRs.

In *S. clavuligerus*, 606 IGRs were conserved, with a homologous region present in at least four strains. While most of these IGRs are conserved in a limited number of strains, it is interesting to note that a significant number of IGRs are present in most of the strains analyzed, making them the “core” IGRs for this group of species (see Figure 2A); among these, 28, 20, and 8 IGRs were found in 37, 36, and 35 species, respectively. It is worth noting that only two of the core IGRs have been previously identified: the 6C RNA, present in 36 genomes, and a glycine riboswitch, preserved in all inspected genomes.

Despite being closely related in the constructed phylogenetic tree, most of the strains belong to different species; thus, the genomic distance from *S. clavuligerus* is greater. This suggests that these IGRs must harbor sequences crucial for the species’ survival. Otherwise, nature would have allowed more mutations in these regions, preventing their detection as homologs.

Moreover, significant differences (Chi-squared test, *p*-value = 4.07 × 10^−15^) were observed between the classification of the conserved IGRs as DT, DP, CO_R, and CO_F (Figure 2B). The presence of sRNAs arising from IGRs flanked by convergent genes, named DT in this study, have been previously reported in *Streptomyces* species in numbers similar to those found here for *S. avermitilis*, *S. coelicolor*, and *S. venezuelae* [6]. Further, our analysis reports that other genetic configurations (CO_R, CO_F, and DP) are better conserved in *S. clavuligerus*.

Within the set of conserved IGRs, RNAz [2] detected 272 putative sRNAs in 245 regions with an RNA-class probability above 0.5; this denotes highly conserved and thermodynamically stable secondary structures. This discrepancy arises because some regions can harbor more than one sRNA. Additionally, in this set, 156 IGRs exhibited the presence of promoter sequences, with 28 containing two promoters and 61 IGRs presenting a terminator sequence. A motif search within these promoters revealed a highly significant motif with an e-value of 1.6 × 10^−72^ (Figure 2C). This motif showed a weak alignment (e-value = 5.58 × 10^−2^) with the VqsR transcription factor (TF) from *Pseudomonas aeruginosa*, suggesting that a different TF may bind to these sequences in *Streptomyces*. What is most striking is that only 19 conserved IGRs have a previous annotation in the RFAM database.

The coding potential analysis revealed that only two conserved IGRs can produce a peptide. The first one is located between the genes CRV15_RS12945 (developmental transcriptional regulator BldC) and CRV15_RS12950 (hypothetical protein) at positions 3,097,871–3,098,267, and it can produce a putative small protein consisting of 113 residues; it is present in 36 of the genomes considered. The other region encodes a small protein of 145 residues and is intriguing, as it contains a che1 RNA motif, which has been reported as a common RNA structure in *Streptomyces*, although its function remains unknown [35].

**Figure 3:**
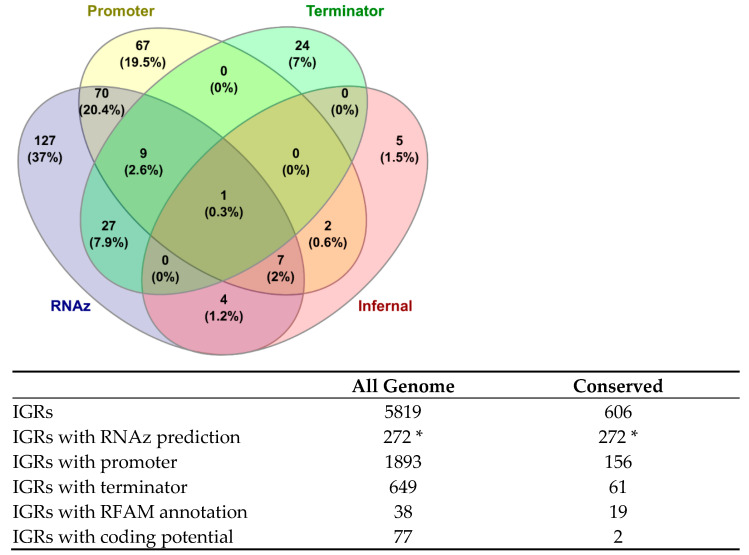
A Venn diagram showing the different features detected in the conserved IGRs. “**RNAz**” indicates an RNA class probability greater than 0.5, suggesting a conserved secondary structure. “**Promoter/Terminator**” designates a promoter or terminator sequence as determined by Promotech or TranstermHP within the IGR, respectively. “**Infernal**” shows the number of known sRNAs annotated in RFAM. The description below the Venn diagram compares the bioinformatics predictions in the conserved IGRs with all IGRs in the genome of *S. clavuligerus.* * RNAz predictions do not differ when considering all IGRs against conserved IGRs because to be detected by RNAz, IGRs must be conserved.

Overall, considering all the features employed in our analyses, it is infrequent to find an sRNA that meets all the criteria (Figure 3). The only one is the IGR flanked by the genes CRV15_RS05425-CRV15_RS05430, which is CO_R, described as an Actino-pnp RNA; this is a structure apparently located in the 5′ untranslated regions of genes predicted to encode exoribonucleases [36]. In this case, the gene CRV15_RS05425 encodes a polyribonucleotide nucleotidyltransferase, a protein with exoRNase activity, confirming that this type of configuration is conserved in *Actinomycetota*.

Conversely, nine conserved IGRs have a promoter, have a terminator, and were detected by RNAz. Interestingly, none of them had been annotated in RFAM; three of them had the promoter and the terminator correctly oriented and are classified as CO_R, suggesting that they can be transcribed as a part of a polycistronic message, while in the other cases, the terminator was inside the promoter or ahead of it.

Among the 19 IGRs annotated in RFAM, 12 were also detected by RNAz. The most common combination of features is the presence of a conserved and thermodynamically stable secondary structure with a promoter, which occurs in 70 instances. The limited number of terminators can be explained by the high %GC content of *S. clavuligerus* IGRs and the terminator motif detected by TransTermHP [37]; it consists of an A-tail followed by a short stem-loop hairpin and a thymine-rich region on their 3′ side [37]. Notably, 60.1% of these terminators are in combination with RNAz predictions.

While information in RFAM for *S. clavuligerus* is limited for Infernal [38] annotations, this database contains 25 entries for riboswitches and cis-regulatory elements and 16 for sRNAs. Of these, 19 were the above-mentioned located in conserved IGRs. The remaining may be asRNAs, which were not addressed in this study, or are structures too lowly preserved across the *Streptomyces* genus to be detected by our conservative approach. Investigating the presence of ncRNAs within IGRs, regardless of their conservation status, we identified 38 ncRNAs from RFAM. This finding is particularly fascinating, as it illustrates that conserved IGRs are enriched for sRNAs, with a ratio of 19 sRNAs out of 606 conserved IGRs, which represents almost five times their occurrence, compared to 38 out of 5819 for all IGRs. In contrast, our analysis of other transcriptional signals in *S. clavuligerus* showed a slightly higher prevalence of promoters (156 out of 606 conserved IGRs vs. 1893 out of 5819 for all IGRs) and terminators (61 out of 606 conserved IGRs vs. 649 out of 5819 for all IGRs) between non-conserved and conserved IGRs (Figure 3).

Based on these findings, we identified conserved regions with RNAz predictions as putative or candidate sRNAs. We opted not to include results from promoters and terminators as features of the sRNAs due to their comparable numbers in both conserved and non-conserved IGRs. When considering all previously known sRNAs available in RFAM, the sensitivity of our detection was 46.3%. This low value can be explained by the fact that we considered only IGRs and not asRNAs, which can drastically reduce the number of detections. Considering uniquely the known sRNAs originating from IGRs, the sensitivity of the method increases to 59.3%. Furthermore, after 1000 executions of the bioinformatics detection, 21 random alignments were classified as having conserved secondary structures by RNAz, resulting in a specificity of 97.9% for our approach.

Our next step was to validate the previously obtained results using RNA-seq data. For this, we constructed a high-resolution transcriptome by incorporating all the data deposited in the SRA database for *S. clavuligerus* along with the libraries generated during the current study.

### 2.2. High-Resolution Transcriptome Assembly

#### 2.2.1. Dataset, Transcriptome Assembly and Expression: sRNAs Have Expression Levels That Are Strongly Dependent on the Specific Environmental Conditions in Which the RNA Was Extracted

We retrieved 70 datasets from the Sequence Read Archive (SRA) [39], corresponding to RNA-seq experiments of *S. clavuligerus* from seven different Bioprojects. Most of the data pertain to RNA extracted at various time points in the growth curve: early exponential, transition, late-exponential, and stationary phases for both the wild-type strain and mutants derived from it, cultured in different media. Additionally, 14 of these samples were generated in our laboratory for mutants of global regulators of CA production in a parallel study [40]. An additional 20 samples were generated during this study; of these, 8 samples correspond to a size-selection library preparation method detailed in Section 4.2.2. In total, we retrieved 1232 GB of data to compile our high-resolution transcriptome. On average, each sample contains 17.5 million reads, with a mean read length of 125 bases (Supplementary File S2).

In total, 14,360 transcripts were assembled using the multiple datasets compiled in this study, with 11,456 transcripts assembled in the chromosome and 2904 corresponding to the plasmid. On average, 82.68% of the reads aligned with the *S. clavuligerus* genome (Supplementary Figure S2). Table 1 summarizes the assembly results, discriminating between the different replicons. All annotated genes were expressed in the transcriptome assembly, covering all complete genes in all cases. Additionally, 7363 transcripts aligned with unannotated regions, and therefore, they were considered predicted RNAs. Out of this high number of novel transcripts, 4913 arise from regions antisense to known genes, while the remaining may be expressed from intergenic regions.

**Figure 4:**
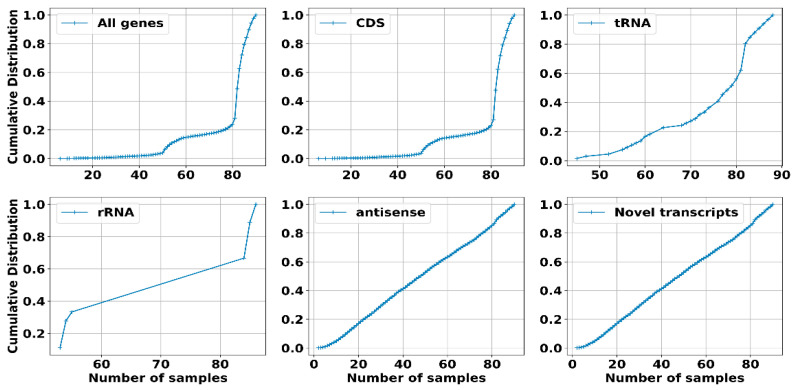
The cumulative distribution of the fraction of transcripts expressed in a given number of RNA-seq samples for all annotated genes. This includes CDS; tRNAs and rRNAs, in addition to non-coding transcripts represented by antisense transcripts; and novel transcripts.

We computed the cumulative distribution to determine whether all genes were expressed in most samples or only in a few. Additionally, we divided the transcripts into five groups to gain more insight into their expression: CDS, tRNA, rRNA, and transcripts corresponding to non-coding RNAs, specifically antisense and novel transcripts, which are likely RNAs arising from intergenic regions (sRNAs) (Figure 4).

Considering all genes, 99% were expressed in more than 30 samples, which was similar to the trend observed for CDS genes, rRNAs, and tRNAs. However, for non-coding RNAs, although most genes were present in many samples, there was a significant proportion of genes expressed in fewer than 30 samples. This reinforces the idea that sRNAs have expression levels that are strongly dependent on the specific environmental conditions in which the RNA was extracted.

**Figure 5:**
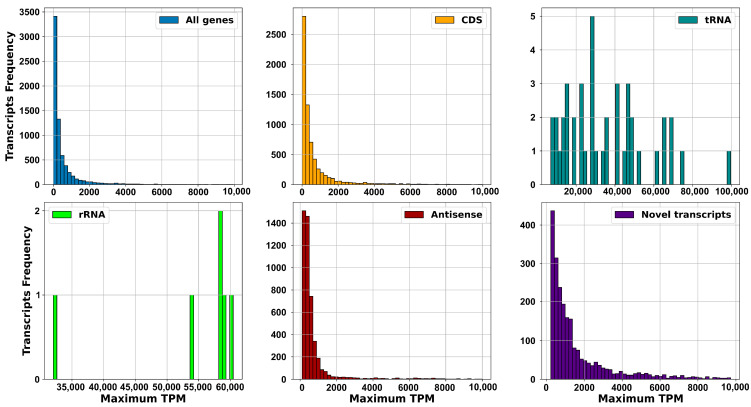
The frequency of expressed transcripts, determined by TPM, in each number of RNA-seq samples for all annotated genes and discriminated by CDS; tRNA and rRNAs, in addition to non-coding transcripts represented by antisense transcripts; and novel transcripts.

Furthermore, we examined the expression levels derived from the raw counts provided by Rockhopper [41] by calculating the transcripts per million (TPM) for each transcript and identifying the maximum value (Figure 5). We maintained the same categorization for the transcripts. As anticipated, tRNAs and rRNAs exhibited markedly higher expression levels, reflecting their essential role in protein synthesis. Conversely, the distribution of expression levels for CDS and non-coding transcripts displayed comparable patterns. Notably, novel transcripts exhibited significantly higher expression levels compared to CDS, underscoring the importance of non-coding RNAs in *S. clavuligerus.*

#### 2.2.2. Operons Prediction: The Multiple RNA-Seq Data Sets Used in This Study, Capturing the Complete Transcriptional Dynamics of *S. clavuligerus*

**Figure 6:**
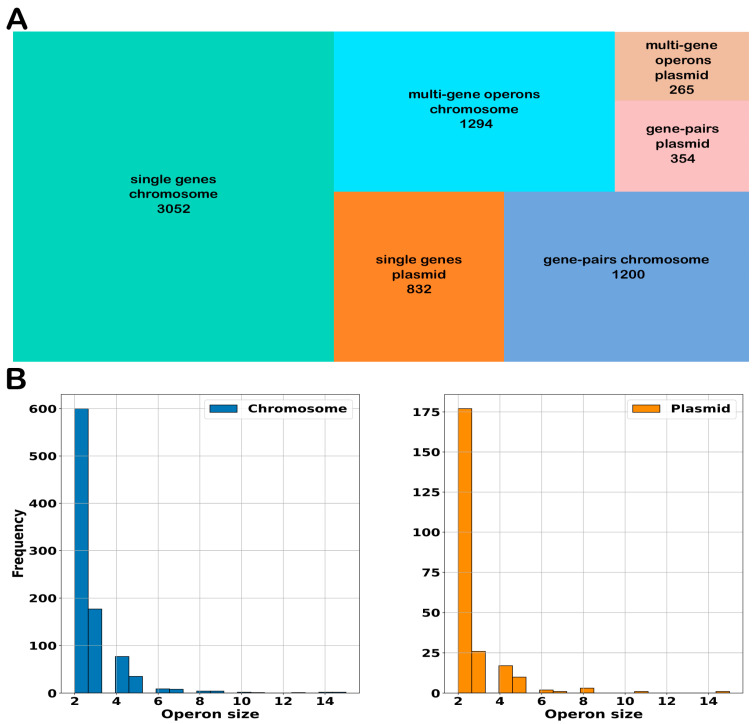
Operons detection by Rockhopper. (**A**) The Treemap showing the number of genes classified as single genes or gene pairs if multi-gene operons in the chromosome or in the plasmid PCLA1. (**B**) The distribution of operon size in the genome of *S. clavuligerus* discriminated by their presence in the chromosome or in the plasmid PCLA1; the *x*-axis represents the number of genes per operon.

Results from Rockhopper showed that 3113 genes were transcribed as part of an operon or transcription unit, representing 44.7% of the total genes annotated in the *S. clavuligerus* genome. Genes were classified according to their expression as single genes, gene pairs, or multi-gene operons by each replicon in the genome, that is, in the chromosome and in the plasmid (Figure 6A). The average number of genes per operon was 4.01 for the chromosome, while for the plasmid, it was 4.34. Interestingly, as shown in Figure 6B, the chromosome contains an operon with 15 genes (ribosomal proteins), two operons with 14 genes (NADH-quinone oxidoreductase and unknown function), and one operon with 13 genes (unknown function). In the plasmid, there are two operons, containing 12 ((−)-δ-cadinene biosynthetic gene cluster) and 11 genes (similarity to depsibosamycin B biosynthetic gene cluster), respectively.

A previous study [30] reported 1648 transcription units (TUs); though they used a multi-omic approach to elucidate the structure of the transcriptome in *S. clavuligerus*, the number of samples was limited, which does not cover all possible scenarios of gene expression, limiting the predictions to the conditions evaluated. Our results surpass the number of TUs reported by these authors because we included multiple RNA-seq datasets, aiming to capture the complete transcriptional dynamics of *S. clavuligerus*.

Supplementary Table S2 shows the largest operons in *S. clavuligerus*, including a description of their functions. We annotated the biosynthetic gene clusters (BGCs) using AntiSMASH v7.1.0 (Supplementary Table S3) [42] and identified the transcriptional units determined by Rockhopper within the BGCs (Supplementary File S3). Of the 44 regions predicted by AntiSMASH, 33 contained operons. The CA cluster is organized into six operons, comprising one multi-gene transcriptional unit of three genes and five gene pairs (Supplementary Figure S3), which is consistent with previous reports [43].

Many other important clusters in *S. clavuligerus* are expressed as sets of polycistronic messages. For example, the cephamycin C cluster contains two multi-gene operons and two gene pairs; the tunicamycin B1 cluster has two gene pairs and a long transcription unit of ten genes; and the holomycin cluster has two multi-gene operons of three and five genes, respectively, along with three gene pairs. Interestingly, the BGC of the clavams has two gene pairs expressed as the same transcriptional message, while the paralogous cluster located in the PCLA1 plasmid contains three of these gene pairs.

#### 2.2.3. Small RNAs Identification: Multiple sRNAs Are Expressed in the Cephamycin C Cluster

To obtain a comprehensive annotation of sRNAs, we combined in silico predictions with high-resolution transcriptome assembly to validate the bioinformatics analysis and elucidate the sRNAs specific to *S. clavuligerus*—those arising from non-conserved IGRs. As shown in Table 1, our RNA-seq data compendium predicted 7363 RNAs. However, due to considerations in the bioinformatics analysis (focusing on conserved IGRs), antisense transcripts were excluded. As a result, the final set comprised 2450 transcripts from intergenic regions, which can be regarded as novel regulatory RNAs; consequently, we focused our analysis on this set.

Due to the possibility that many transcripts could represent transcriptional noise and false positives, we applied a filtering step to retain only predicted RNAs detected in at least 10 RNA-seq samples. Additionally, we applied an extra filter to include only those with a minimum of 20 reads aligned per sample. As a result, from the initial set of novel transcripts, 921 transcripts were retained as our final set of reliable predicted sRNAs, as demonstrated by their substantial expression. It is noteworthy that these transcripts originated from 825 IGRs, indicating that some IGRs can encode more than one sRNA, which are comparable with the in silico predictions of RNAz.

In addition to the 921 transcripts identified as predicted RNAs, we used APERO [44] to generate and analyze eight size-selected, paired-end RNA-seq libraries to detect small RNAs expressed at four points of the growth curve of *S. clavuligerus* during CA production [44]. Samples were collected at four distinct time points (see Section 4—RNA Extraction and Sequencing). APERO detected 256 intergenic sRNAs, of which 83 overlapped with Rockhopper predictions (see Supplementary Figure S4). A new GFF file was generated, encompassing annotations from both software tools, which was subsequently employed for a differential expression analysis.

Out of the 606 conserved IGRs in *S. clavuligerus*, 203 exhibited some level of expression. This proportion of conserved IGRs with detectable expression is significantly higher than that observed for all IGRs in the genome (825/5976). Furthermore, the conservation of secondary and thermodynamically stable structures has proven to be a reliable feature for sRNA detection. This was evidenced by the number of annotations from RFAM retrieved in the in silico prediction and the substantial number of non-coding RNAs identified by RNAz (123/245) that are validated by RNA-seq data.

Among the ncRNAs annotated in RFAM, 19 were found to be expressed under the conditions included in the transcriptome assembly. As in this study, we prioritized specificity over sensitivity; our strict filtering criteria discarded some sRNAs, as demonstrated by the absence of expression for the 6C RNA, which was detected when no restrictions were applied to the number of reads aligned to a transcript.

To evaluate our current approach, we compared our predictions with the transcription units reported in Hwang et al. (2021) [30]. As previously mentioned, our methodology recovered many more operons and non-coding RNAs. We used their results to assess the precision of our genomic coordinate predictions for the sRNAs, which is a major limitation in this type of study. Their methodology included dRNA-seq and term-seq, techniques that have demonstrated high precision and reproducibility in identifying transcription start sites (TSSs) and transcript 3′-end positions (TEPs).

Considering the premature and intergenic TU, which amount to 150 and 39 TUs, respectively, we discovered 63 pre-TUs and 14 intergenic TUs in our dataset. We then compared the TSSs and TEPs and found that on average, our method differs by 39 nucleotides for TSSs and 50 nucleotides for TEPs. The existence of multiple alternative sites for transcription initiation in different experimental conditions [45] or different transcripts arising from the same intergenic regions complicates the precise determination of the genomic location of non-coding RNAs. Nevertheless, our method was a reasonable approach given the genome-wide nature of the study and its scope, which was to demonstrate the wealth of ncRNAs encoded in *S. clavuligerus* genome.

Finally, we observed the incidence of sRNAs in the previously described operons, with 87 predicted sRNAs being transcribed as part of a transcriptional unit. Notably, none were detected in the BGC for CA production or in the paralogous cluster for clavams synthesis located in the plasmid PCLA1. In turn, in the alanylclavam cluster, a weak expression of an sRNA was observed in the middle of the gene pair CRV15_RS13875-CRV15_RS13880, present in 49 samples with an average of 71 TPM.

**Figure 7:**
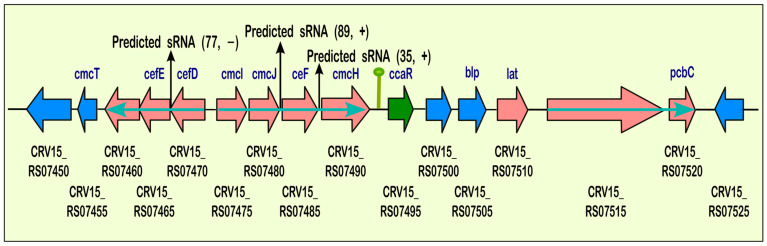
The operon structure of the BGC for cephamycin C. Sites where predicted sRNAs arise are represented by black arrows; Parenthesis: length and strand. Genes are represented as horizontal thick arrows. TU are depicted as genes bound together by a light blue arrow. Green: transcriptional regulator; pale red: biosynthetic genes; grey: other genes; blue: transport-related genes; vertical green line: binding site.

The most notable result was the expression of multiple sRNAs in the cephamycin C cluster. Here, we detected the expression of an sRNA in the TU (CRV15_RS07460, CRV15_RS07465, CRV15_RS07470) at the position 1868861-1868784 on the reverse strand and two sRNAs in the TU (CRV15_RS07475, CRV15_RS07480, CRV15_RS07485, cmcH), both on the forward strand with genomic locations 1872525-1872614 and 1873578-1873613, respectively. Figure 7 summarizes this transcriptional organization detected in this BGC.

No other features, such as the promoter, terminator, conservation, secondary structure, or coding sequence, were detected for these sRNAs in the cephamycin C biosynthetic cluster. The absence of a promoter sequence for these sRNAs suggests that they may result from the processing of the 5′ or 3′ UTRs of their flanking genes [46]. Additionally, they can be considered specific to *S. clavuligerus*, as their sequences show no conservation in other Streptomyces species.

It is remarkable that no expression was observed from the IGRs between the genes *cmcH-ccaR*, which was reported to influence the production of cephamycin C [47]. Therefore, the regulation of this region is not driven by sRNA and could be given by the presence of transcriptional signals, e.g., binding sites of transcription factors.

#### 2.2.4. Differential Expression: sRNAs Can Act as Global Regulators in *S. clavuligerus*

**Figure 8:**
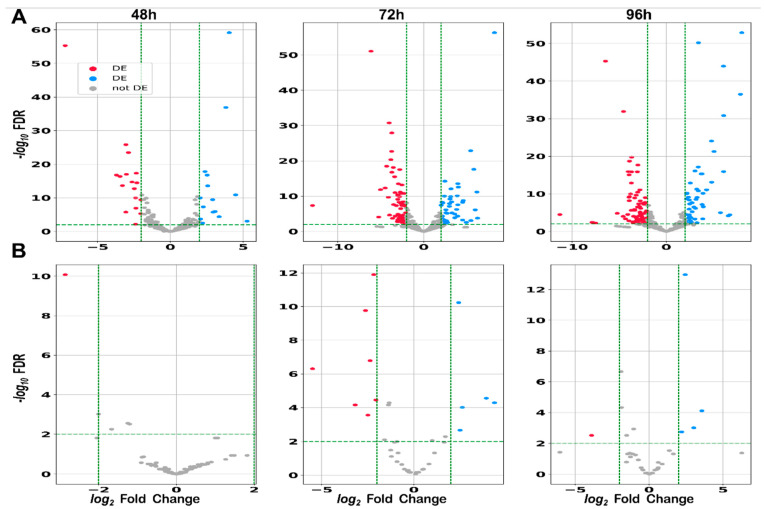
The volcano plot for DE sRNAs during the production of CA at different time points for (**A**) the chromosome and (**B**) the plasmid PCLA1 of *S. clavuligerus*. DE sRNAs have a log2FC >= 2 and an FDR < 0.01. Blue: upregulated sRNAs; red: downregulated sRNAs.

Following the identification of sRNAs encoded in IGRs, we analyzed their differential expression during the growth of *S. clavuligerus* and CA production. As previously mentioned, we collected samples at four time points (24, 48, 72, and 96 h), which represent key stages of growth, corresponding to different levels of CA production (see Supplementary Figure S5). For the differential expression analysis, the 24 h sample was used as the reference point due to the low levels of CA observed at this stage. We then compared gene expression at the remaining time points against this baseline. Our focus was particularly on the 96 h time point, as this is when the strain reaches the maximum CA production. The results are presented in Figure 8 as volcano plots for the three time points, distinguishing between the two replicons of the *S. clavuligerus* genome: the chromosome (Figure 8A) and the large plasmid PCLA1 (Figure 8B).

Overall, for the differentially expressed (DE) genes, both the magnitude (measured as Log2 fold-change) and the number of significant DE genes were greater at 72 and 96 h compared to 48 h (Figure 8). This indicates that the effects on gene expression were more pronounced and numerous at the later time points for both upregulated and downregulated genes. This observation is coherent, as the morphological differentiation reached by the strain at 48 h is not as meaningful as that at the other two points [48]. Supplementary File S4 contains the detailed results of the differential expression analysis.

To understand how these sRNAs exerted influence on CA biosynthesis, predictions of RNA–RNA interactions (DE sRNAs identified at 96 h of cultivation with their potential mRNA targets) were carried out using IntaRNA 2.0 [49] and TargetRNA3 [50]. Only interactions with a hybridization energy less than −20 kcal/mol and with a probability above 0.5 were considered. The sRNA–mRNA interactions showed that sRNAs can act as global regulators, as most of the sRNAs have many mRNA targets. This is because trans-RNAs, the focus of this study, have loci in the IGRs, allowing them to have partial complementarity with mRNAs and to interact with multiple RNAs simultaneously (Supplementary Figures S6–S11).

**Figure 9:**
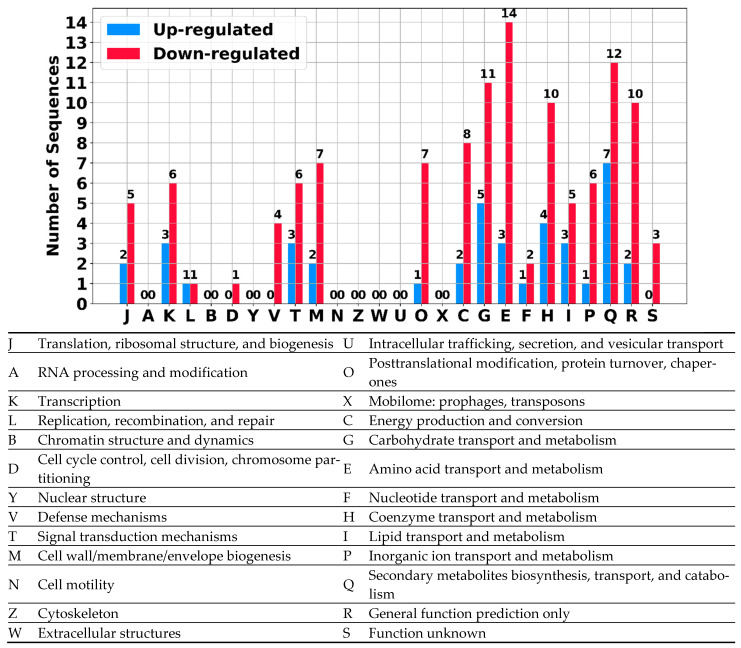
Cluster of Orthologous Groups (COG) functional classification for gene products from mRNAs potentially interacting with the differentially expressed (DE) sRNAs at 96 h.

To identify the processes altered by the differentially expressed (DE) sRNAs during CA production, we annotated the genes potentially interacting with these sRNAs. First, we classified the genes using the Cluster of Orthologous Groups (COG) database https://www.ncbi.nlm.nih.gov/research/cog accessed on October 2024 (Figure 9, Supplementary Figures S12 and S13). Notable differences were observed across almost all categories. For example, at 96 h, the categories C, G, E, and Q were over-represented among the downregulated genes, suggesting that sRNAs influence carbohydrate and amino acid metabolism, energy production, and secondary metabolite biosynthesis.

To provide a detailed description of the metabolic processes affected by the sRNAs, we used KofamKoala [51] to obtain the KEGG terms for these genes and then used the KEGG Mapper Reconstruction tool https://www.genome.jp/kegg/mapper/reconstruct.html accessed on 1 October 2024. The analysis revealed that the biosynthesis of secondary metabolites was the process most affected by sRNAs at the 96 h time point, specifically terpenoid biosynthesis, followed by amino acid metabolism and the biosynthesis of cofactors. Furthermore, at 72 h, genes related to CA or cephamycin C biosynthesis did not appear in the KEGG annotations, suggesting that sRNAs involved in their regulation are not active at this time point. Interestingly, at 48 h, secondary metabolism is not over-represented in either upregulated or downregulated genes.

A detailed analysis of the IntaRNA and TargetRNA3 results revealed that one of the predicted sRNAs (“p_RNA_R_5138795.0-5139023.0+”) may interact with an mRNA in the CA cluster. This mRNA corresponds to the gene CRV15_RS07535, which encodes (carboxyethyl)arginine beta-lactam-synthase, an enzyme catalyzing one of the initial steps in CA biosynthesis. Regarding the cephamycin BGC, the *lat* gene appears to be the target of an sRNA upregulated at 96 h (“p_RNA_R_3405987.0-3406553.0+”), while the *cefE* gene is targeted by the sRNA “p_RNA_A_1867161-1867625”, which is downregulated at the same time point.

The above-mentioned sRNAs share several common features: none of them are conserved, they do not contain terminator or promoter sequences, and they are not part of an operon structure. Additionally, all these sRNAs interact with multiple mRNAs, suggesting their role as global regulators in *S. clavuligerus*. Since they originate from intergenic regions (IGRs), they do not exhibit full complementarity with mRNAs, which explains why a single sRNA can regulate many mRNAs.

**Figure 10:**
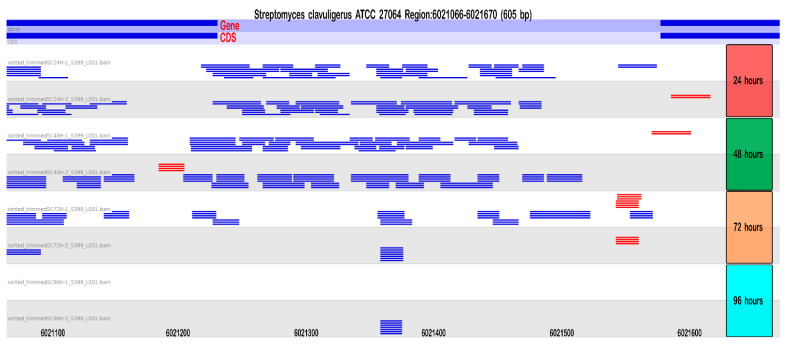
The read alignment of the sRNA “p_RNA_R_6021554-6021232-” to the genome of *S. clavuligerus* at different time points considered in this study. The “p” stands for predicted; “R” indicates the prediction comes from Rockhopper. In the sRNA name, “6021554-6021232” are the genomic coordinates, and “-” at the end denotes the strand. The alignments were visualized in SeqMonk. Blue and red represent the reverse and forward strands, respectively.

Additionally, we investigated whether any interactions between the DE sRNAs and known regulators of the CA production cascade were shared, e.g., *adpA*, *bldG*, *relA*, *areB*, and *brp*. Among these, only *relA* showed interaction with “p_RNA_R_6021554-6021232-”, which is predominantly expressed during the initial growth phase of *S. clavuligerus*. These predictions were reinforced by an opposite expression (OE) analysis, which is based on the principle that if the sRNAs are upregulated, their target mRNAs will be downregulated and vice versa [52]. The alignment of the reads to the regions from which this RNA comes from is shown in Figure 10 to illustrate the variation in its gene expression during cell growth.

Finally, as the COG functional annotation revealed that amino acid metabolism was one of the processes most influenced by sRNAs, we focused on the metabolism of arginine because it is a precursor for CA synthesis. Arginine is part of the regulon of “p_RNA_R_6021554-6021232-” and “p_RNA_R_3405987-3405553+”, the sRNA described earlier that showed interactions with the BGC of cephamycin C. Additionally, two other sRNAs, “p_RNA_R_4510526-4510667+” and “p_RNA_R_4703341-703077-,” target the expression of genes in the arginine biosynthesis metabolic pathway. A summary of all the results from IntaRNA, TargetRNA3, and OE is available in Supplementary File S5.

## 3. Discussion

Species of the *Streptomyces* genus remain a rich source of novel bioactive compounds, as revealed by multiple genome mining studies [53,54]. This is of vital importance to the pharmaceutical industry, particularly in the search for new antibiotics, as microbial resistance is rapidly increasing worldwide.

Numerous strategies have been implemented to enhance the production of known compounds and to discover novel ones. These strategies include optimizing media and culture conditions, using different bioreactor configurations [55,56], heterologous expressing biosynthetic gene clusters in chassis strains optimized for rapid growth and secondary metabolite production [57,58], introducing transcriptional regulators both individually and in families [59], and overexpressing genes involved in the metabolic pathways for secondary metabolite production [60].

Despite extensive efforts targeting diverse metabolic and regulatory pathways through genetic engineering, the non-coding regions of the genome remain a largely unexplored area. These regions harbor numerous regulatory RNAs, which have been shown to play crucial roles in other prokaryotes [61]. As the number of regulatory functions attributed to these RNAs has increased, it is now clear that they are directly involved in the production of secondary metabolites [62].

Since there is no straightforward method to identify these genes [63], the first step towards understanding their roles is a comprehensive annotation and characterization of their genomic locations, structures, interactions with other molecules, and the dependence of their expression on culture phases, the organism’s life cycle, or environmental stresses that trigger their biosynthesis. This foundational knowledge is essential for any future genetic modifications involving ncRNAs. We pursued this task using a traditional bioinformatics approach and subsequently refined the predictions using a compendium of RNA-seq data to generate a high-resolution transcriptome. Our goal was to encompass all possible expression patterns that could be present in *S. clavuligerus*.

It is noteworthy that a substantial number of IGRs are conserved in *S. clavuligerus*, hinting at the presence of functional elements encoded within these IGRs. This observation is supported by Tsai et al. (2015) [23], who demonstrated that conserved IGRs are enriched for ncRNAs. Although we attempted to determine the pan-genome of the *Streptomyces* genus in terms of the IGRs [34], we found that none of these regions are conserved across the entire genus. Instead, it appears that IGRs are specific to the clades in which *Streptomyces* is divided [64], and many IGRs and sRNAs are strain-specific. The identification of conserved IGRs in *S. clavuligerus* exhibited similar behavior, with 606 IGRs showing sufficient similarity to be considered homologous in other *Streptomyces* strains.

We prioritized specificity over sensitivity to avoid false positives and to ensure trustworthy predictions. Therefore, requiring conservation in at least four strains served as a strong filter, even if it meant discarding less conserved IGRs (i.e., those with less than 70% similarity or sequences present in fewer strains but still conserved). This decision was also influenced by the fact that RNAz provides better predictions when more sequences are used for alignment [2].

Regarding transcriptional signals, we and previous studies hypothesized that for an sRNA to be expressed, it must have a promoter sequence where a sigma factor can bind and a terminator sequence to end the transcription process [65,66].

While TranstermHP predictions may not reliably indicate the existence of an sRNA, the promoters identified by Promotech, although evenly distributed across the genome, provided some insight into the promoter sequences that drive the expression of certain sRNAs. As mentioned earlier, TranstermHP’s performance can be limited by the high GC content of Streptomycetes. It has been proposed that a different terminator motif or a different termination mechanism, such as Rho-dependent termination, may be predominant in Streptomycetes [21].

In the case of promoter sequences, though they are not particularly enriched in conserved IGRs, we identified a motif shared by all promoters within IGRs. However, the transcription factor that binds this sequence remains to be elucidated. Notably, some promoter sequences were located exactly one nucleotide upstream of the transcription start site (TSS) determined for certain pre-TUs by Hwang et al., 2021 [30], indicating their role in the expression of sRNAs in these bacteria. Finally, while Promotech’s predictions could be improved with the inclusion of more known promoters from actinomycetes in its training, it represents a valuable tool for understanding the regulatory landscape of sRNAs in *S. clavuligerus*.

Because RNAz detected 12 out of the 19 sRNAs annotated in RFAM that are present in the IGRs of *S. clavuligerus*, this method has proven its ability to predict reliable putative sRNAs. However, the main shortcoming of RNAz is its inability to determine the exact coordinates of the ncRNAs, as the detection depends on the multiple sequence alignment (MSA), which can extend beyond the precise locations. We surpass this limitation with pre-processing with rnazWindow.pl, which allowed for the exploration of longer regions and complement bioinformatic predictions, as IGRs did not have to be restricted to a specific number of base pairs. The requirement for a MSA highlights another limitation of RNA-based analyses: specific ncRNAs cannot be detected because this approach relies on sequence conservation.

To our knowledge, this is the first attempt to characterize the complete transcriptome of *S. clavuligerus* using multiple datasets during the assembly process. As a result, we obtained a map of all produced transcripts, which included all annotated genes and novel transcripts, many of which were sRNAs encoded in intergenic regions, validating our in silico predictions.

The transcriptome assembly revealed that all annotated genes were expressed, including every complete gene in the dataset. Notably, this includes all genes associated with the biosynthetic gene clusters (BGCs) in *S. clavuligerus*, indicating that this bacterium can produce a range of uncharacterized compounds. The expression of these genes across diverse experimental conditions, particularly in the wild-type strain, strengthens this notion.

To substantiate these findings, we calculated the cumulative distribution, maximum TPMs, and the average number of samples in which each BGC was expressed (Supplementary Figures S14 and S15). The results indicated that secondary metabolism is broadly active under the conditions tested. This discovery opens the exciting possibility of harnessing *S. clavuligerus* to produce novel bioactive compounds, as its ability to express the necessary genes for their biosynthesis remains intact. Further in-depth analyses of the RNA-seq data could reveal the specific conditions under which certain BGCs are expressed, offering valuable insights for future biotechnological applications. This finding is a breakthrough, offering a promising avenue for discovering and producing new bioactive compounds from a well-known organism.

Although it was not explored in-depth, considering the initial objectives of the study, the 4913 antisense transcripts, representing 34.21% of the expressed transcripts, demonstrate that this process is also extensive in Streptomycetes, similar to what has been shown for other bacteria [67]. These outcomes indicate that the transcriptome in *S. clavuligerus* is extremely complex, and the elucidation of all expression patterns is far from being fully understood.

A full description of the operon structure has not been reported for *S. clavuligerus*. Therefore, the results obtained in this study are valuable for the community involved in CA and secondary metabolite biosynthesis in this bacterium. Since genes that are co-transcribed frequently have related functional roles or are involved in the same metabolic pathways, identifying multi-gene transcription units can shed light on the functional relationships and co-regulation of genes [68].

Predicted sRNAs displayed distinctive expression patterns under the tested conditions. Their interaction networks extend to almost all biological processes within the cell, with some sRNAs potentially acting as master regulators due to their interactions with multiple targets, similar to findings in other bacteria [65]. Notably, the main processes affected by sRNAs appeared to be the biosynthesis of secondary metabolites (See Figure 9) Most of these sRNAs are unknown and do not exhibit conservation across the *Streptomyces* genus, suggesting that they are specific to *S. clavuligerus*. Future studies should focus on understanding the specific roles of these sRNAs in detail.

We investigated the differential expression of sRNAs during CA production and identified two sRNAs predicted to interact with genes of the CA BGC based on a bioinformatic analysis using IntaRNA and TargetRNA3. These two sRNAs, “p_RNA_R_6021554-6021232-” and “p_RNA_R_5138795.0-5139023.0-”, are proposed to act as global regulators and represent good candidates for metabolic engineering studies, particularly by deleting the intergenic regions that contain them. For instance, “p_RNA_R_6021554-6021232-” is absent during the peak production time of CA (Figure 10), suggesting it negatively regulates clavulanic acid production. The proposed mechanism, based on our predictions, suggests that this ncRNA may block the early steps of CA synthesis and other processes by interacting with the global regulator *relA*. This effect may be supported by the activity of other sRNAS, such as “p_RNA_R_5138795.0-5139023.0-”, which is predicted to inhibit the production of (carboxyethyl)arginine beta-lactam synthase. However, once these ncRNAs are no longer expressed—as indicated by their lack of expression at 72 and 96 h—the predicted de-repression of their target genes might allow for the synthesis of CA. Therefore, their inactivation could potentially lead to increased CA production. The secondary structure of “p_RNA_R_6021554-6021232” is depicted in Supplementary Figure S16.

Furthermore, sRNAs potentially influence the metabolism of amino acids, which has direct implications for CA production. CA synthesis requires a strong demand for nitrogen-derived precursors, intensifying nitrogen metabolism when antibiotic production starts [69]. Amino acids are essential for CA biosynthesis, as many of them (arginine, ornithine, aspartate, glutamate) are precursors of CA. Thus, the presence of multiple amino acids provides more suitable precursors for CA by reducing the demand for C-3 compounds required for anaplerotic reactions [70,71].

Overall, the findings of this study can be summarized as follows: the *S. clavuligerus* genome harbors a rich diversity of ncRNAs, including both sRNAs and asRNAs, as demonstrated by their expression across multiple datasets. Furthermore, the transcriptomic dynamics of *S. clavuligerus* reveal a strong dependence of sRNA expression on both time and culture conditions.

The results presented here provide valuable insights into these expression patterns, and the predictions are now available for the scientific community to explore. Understanding the expression patterns of multiple sRNAs could significantly contribute to uncovering the post-transcriptional regulatory mechanisms involved in various metabolic processes, with a particular focus on secondary metabolite production.

This situation is not unique to *S. clavuligerus*. For instance, in *Bacillus subtilis*, more than 100 ncRNAs have been identified through transcriptomic studies, yet only 15 have been characterized in detail, and most of their targets remain unknown [72]. Thus, the ncRNA world in *S. clavuligerus* is still largely uncharted and awaits further exploration.

## 4. Materials and Methods

In general terms, we performed a comparative genomic analysis of the IGRs in *S. clavuligerus* against closely related strains, a transcriptome assembly to obtain a comprehensive view of the *S. clavuligerus* transcriptome, and a differential expression analysis of the annotated sRNAs to identify their relationship with CA production.

### 4.1. In Silico Prediction of Small RNAs

#### 4.1.1. Intergenic Regions Extraction

We selected 36 genomes of Streptomycetes belonging to the clade where *S. clavuligerus* is located in a phylogenetic tree constructed in our previous study using 633 core genes retrieved from 121 genomes of *Streptomyces* species [34]. Using custom Biopython v1.81 scripts, we extracted IGRs for each genome, defining IGRs as sequences between genes annotated as CDS, tRNA, or rRNA. To avoid any bias, we did not trim the IGRs at their extremes, as the location of the sRNAs from the neighboring genes can be variable. Previous annotations of non-coding RNA were included in our analysis and utilized as positive control. Accession numbers for genome assemblies used in the IGR comparison are listed in Supplementary Table S1.

#### 4.1.2. Conservation Analysis of IGRs

We assessed the conservation of IGRs using reciprocal best BLAST hit (RBBH) analysis between the IGRs in the genome of *S. clavuligerus* and those of the other selected *Streptomyces* species. Hits with an e-value less than 1 × 10^−5^, with query coverage of more than 70%, and present in at least four species were deemed conserved and utilized for subsequent analysis.

#### 4.1.3. Detection of Structured Non-Coding RNAs and Transcriptional Signals

For each group of conserved IGRs, a multiple sequence alignment (MSA) was performed using Clustalw2 v2.1 [73] with default parameters. The MSAs were utilized by RNAz v2.1 [2] to identify RNAs with conserved and thermodynamically stable secondary structure; the following programs from the RNAz suite were employed: first, rnazWindow.pl --opt-id = 70 -max-length = 200, and subsequently RNAz --both-strands -*p* 0.5. In all cases the IGR sequence of *S. clavuligerus* served as the reference. The presence of promoters and terminators in the IGRs was determined by Promotech [74] and TransTermHP v2.09 [37], respectively. Motifs of promoter sequences found in the conserved IGRs were discovered with MEME v5.5.5 and annotated with Tomtom [75].

#### 4.1.4. Search for Conserved IGRs in the RFAM Database

The conserved IGRs of the *S. clavuligerus* genome were annotated against the RFAM database [76]. Initially, we aligned them using BLASTN v2.16.0 with default parameters. Subsequently, we employed the cmscan program from the Infernal 1.1.5 package [38]. The coding potential for peptide production was calculated using CPC2 v1.0.1 [77] for all identified IGRs.

Finally, all the results from the conservation analysis, promoter and terminator prediction, secondary structure thermodynamic stability assessment, BLAST and Infernal annotations, and coding potential evaluation were combined and summarized in tabular format using in-house Python scripts (Supplementary File S1).

#### 4.1.5. Statistical Analysis

To estimate the specificity of our approach, the preceding methodology was replicated and executed 1000 times, creating random sequences from all the IGRs of *S. clavuligerus* and counting the number of IGRs with a thermodynamically stable secondary structure according to RNAz. The sensitivity of the method was determined by the number of sequences annotated in RFAM in the final set of conserved IGRs and detected as ncRNAs by RNAz.

### 4.2. High-Resolution Transcriptome Assembly

#### 4.2.1. Bacterial Strains and Culture Conditions

*S. clavuligerus* ATCC 27064 mycelium was stored at −80 °C in a glycerol solution (20% *v*/*v*). Composition of the seed medium for mycelia growth was as follows (g/L): glycerol, 15; peptone, 10; malt extract, 1.0; MgSO_4_, 0.75; MnCl_2_, 0.0001; FeSO_4_, 0.001; ZnSO_4_ 0.001; MOPS, 21; and K_2_HPO_4_, 0.8. For CA production, the following culture medium was employed: soy protein isolate (ISP) medium (g/L): glycerol, 30; soy protein isolate, 25; K_2_HPO_4_, 0.8; MgSO_4_·7H_2_O, 0.75; MnCl_2_·4H_2_O, 0.0001; FeSO_4_·7H_2_O, 0.001; ZnSO_4_·7H_2_O, 0.001; MOPS, 21.0; and pH fixed at 6.8 [48]. The pre-culture medium had the same composition as that of the culture medium except for glycerol concentration, which was adjusted to 15 g/L. All *S. clavuligerus* cultures were carried out in 250-baffled Erlenmeyer flasks containing 50 mL of medium. Pre-culture flasks were inoculated with seed medium (10% *v*/*v*), and the cultivation medium was inoculated with 10% *v/v* of pre-culture medium. Cultures were performed in triplicate and incubated for 120 h at 220 rpm and 28 °C.

#### 4.2.2. CA Determination

For sampling, 2 mL aliquots were taken from each flask every 24 h during the fermentation process (24, 48, 72, and 96 h). For CA quantification, the culture samples were centrifuged at 14,000× *g* for 10 min at 4 °C and filtered (0.22 µm). CA was determined by HPLC Agilent 1200 (Agilent Technologies, Waldbrom, Germany) equipped with a Diode Array Detector (Agilent Technologies, Palo Alto, CA, USA) at 312 nm using a reverse phase ZORBAX Eclipse XDB-C18 (4.6 mm × 150 mm, 18 µm Agilent Technologies, Palo Alto, CA, USA) column; 94% *v/v* KH_2_PO_4_ (50 mM, pH 3.2) and a 6% *v/v* methanol solution were used as mobile phase at 1 mL/min. CA was imidazole derivatized at a ratio of 1:3; the reaction was kept at 28 °C for 15 min [78].

#### 4.2.3. RNA Extraction and Sequencing

Biomass samples, consisting of 2 mL of culture medium, were collected at the same time points as those used for CA determination, with the latter marking the point of maximum CA production. Duplicate samples were taken and centrifuged at 10,000× *g* for 15 min. TRI Reagent^®^ (Sigma-Aldrich, St. Louis, MO, USA) was then added, following the manufacturer’s instructions, to preserve RNA integrity. The cell pellets were immediately stored at −80 °C until they were prepared for sequencing. Total RNA extraction was performed using the Omega Bio-Tek Mag-Bind^®^ (Norcross, GA, USA) kit, followed by two rounds of DNase I treatment and purification using the Monarch^®^ (New England Biolabs, Ipswich, MA, USA) RNA Cleanup kit for RNA cleanup and concentration. The RNA purity and concentration were assessed using the Agilent 4150 TapeStation (Santa Clara, CA, USA) and Thermo Scientific™ NanoDrop™ (Waltham, MA, USA). Library preparation was performed with the NEXTFLEX^®^ Small RNA Sequencing Kit V4 (Waltham, MA, USA), and RNA sequencing was performed on a HiSeq X Ten Illumina platform with 2 × 150 bp paired-end (PE) reads.

For total RNA sequencing, the cell samples were grown and harvested as described previously at the same time points. RNA was extracted from three biological replicates using the Qiagen RNeasy Plus Universal Mini Kit (QIAGEN, Hilden, Germany). RNA samples were quantified using the ThermoFisher Scientific™ Qubit 2.0 Fluorometer, and RNA integrity was checked using the Agilent 4200 TapeStation. Samples were initially treated with TURBO DNase (Thermo Fisher Scientific, Waltham, MA, USA) to remove DNA contaminants. The next steps included performing rRNA depletion using QIAseq^®^ FastSelect™—rRNA Bacteria (QIAGEN, Hilden, Germany), following the manufacturer’s protocol. Strand-specific RNA sequencing libraries were prepared using the NEBNext Ultra II Directional RNA Library Prep Kit (New England Biolabs, Ipswich, MA, USA) for Illumina according to the manufacturer’s instructions. The samples were sequenced using a 2 × 150 bp PE configuration on an Illumina HiSeq 2500 platform.

#### 4.2.4. Data Collection

The Bio.Entrez Programming Utilities from the Biopython package v1.81 were employed to retrieve accession numbers for SRA experiments related to *S. clavuligerus* and the associated metadata. Filters were applied to include only samples with an RNA-seq library strategy and a transcriptomic library source. FASTQ files were downloaded from the Sequence Read Archive (SRA) [39] using the SRA Toolkit version 3.1.0 with the fasterq-dump tool, resulting in 70 downloaded datasets. Additionally, 20 samples were generated during this study, as described in the previous section—8 for sRNA sequencing and 12 for total RNA sequencing. In total, we compiled 90 RNA-seq datasets.

#### 4.2.5. Transcriptome Characterization

To characterize the transcriptome structure of *S. clavuligerus*, we utilized Rockhopper v2.0.3 with default parameters [41]. Rockhopper internally aligns reads, performs transcriptome assembly, identifies operons, and detects novel RNAs. Acknowledging that sRNA expression can arise from noise signals [79], we filtered the results to include only putative RNAs detected in at least 10 RNA-seq libraries. Additionally, we applied a further filter to retain only those with a minimum of 20 reads aligned per sample. While our primary focus was on sRNAs originating from IGRs, antisense predictions were not analyzed in this study, although we plan to consider them in future research.

### 4.3. Expression Profile of sRNAs During CA Production

#### 4.3.1. Small RNA Detection by APERO

To complement sRNAs predictions, we implemented APERO [44], focusing specifically on detecting sRNAs directly involved in CA production. For this purpose, we utilized the samples generated during the growth of *S. clavuligerus* for CA production (refer to Section 4.2.3). The predictions from each software were merged, and the combined results were labeled as putative sRNAs.

To prepare the inputs for APERO, the raw reads underwent trimming and quality filtering using fastp v0.24.0 [80]. Quality control checks were conducted using FastQC v0.12.1, and the results were summarized using MultiQC v1.25.2. Subsequently, the reads were aligned using Bowtie2 v.2.5.4 [81]. Samtools v1.21 [82] was employed to convert SAM files to BAM format, sort the BAM files by alignment position, and generate indexed BAM files.

#### 4.3.2. Differential Expression

A GFF file containing putative sRNAs predicted by Rockhopper and APERO was generated using in-house Python scripts v.3.8 (refer to ‘Code availability’ section). This GFF file was then used alongside the BAM files to run HTSeq-count [83]. The following command was executed: htseq-count -f bam -t putative_sRNA -i name -n 20 -r pos -bam_file file_gff.

Finally, we obtained a set of DE sRNAs during CA production using PyDESeq2 v0.4.12 [84]). We compared the expression patterns throughout the growth curve of *S. clavuligerus*, starting from the adaptive phase (24 h), where low production of CA is observed, through the exponential growth phase (48 and 72 h) and up to the maximum production of the metabolite (96 h).

#### 4.3.3. Small RNAs Interactome

IntaRNA 2.0 was utilized to predict sRNA–mRNA interactions [49]. The IntaRNAsTar mode was activated, which incorporates optimized parameters for genome-wide sRNA target prediction. For this analysis, the sequences of DE sRNAs longer than 50 nucleotides in each condition studied were used against all coding sequences annotated for *Streptomyces clavuligerus*. Due to the limited availability of information regarding transcription start sites (TSSs) and terminator sequences for this species, these features were not included in the analysis. The interaction search was allowed to cover any region within the coding sequence without restrictions to proximity to the start or stop codon. The primary constraint imposed by the IntaRNAsTar personality was the maximum interaction length of 60 bases. By analyzing the targets identified by multiple programs employing diverse approaches, we aim to enhance the reliability of the predicted sRNA–target interactions. Thus, we also included the predictions of the tool TargetRNA3 https://github.com/btjaden/TargetRNA3/tree/main accessed on 1 November 2024 [50] with default parameters. Only targets with an RNA–RNA interaction energy of less than −20 kcal/mol and with a probability of 0.5, were considered for further analysis. Cluster of Orthologous Groups database [85] and KEGG orthologs assignment [51] were used to annotate mRNAs that showed interaction with the DE sRNAs across all the conditions tested. In addition, DE sRNAs’ secondary structures were visualized with RNAfold [86].

#### 4.3.4. Total RNA Analysis

The bioinformatics workflow for total RNA analysis was similar to the one employed for the sRNA DE analysis, that is, fastp v0.24.0, FASTQC v0.12.1, MultiQC v1.25.2, Bowtie2 v2.5.4, Samtools v1.21, HTSeq-count v2.0.3, and PyDESeq2 v0.4.12. In addition, before read alignment, residual ribosomal RNA was removed with SorteMeRNA v4.3.7 [87]. With the information of DE mRNAs (Supplementary File S6), the opposite expression (OE) was performed: mRNAs and sRNAs with a log2 fold change ≥ 2 and a corrected *p*-value ≤ 0.01 were classified as upregulated, while those with a log2 fold change ≤ −2 and a corrected *p*-value ≤ 0.01 were classified as downregulated. An mRNA exhibiting opposite expression to an sRNA in at least two differential expression analyses was identified as a potential target of that sRNA.

#### 4.3.5. Code Availability

All analyses presented in this work were conducted on a Dell Precision 5820 WorkStation equipped with an Intel Xeon W-2255 processor, 10 cores, and 128 GB of RAM memory. The code used for data processing, generating summary tables, and creating figures is available at https://github.com/CarlosCaicedoM/smallRNAs_Sclav/ (accessed on 3 May 2023).

## 5. Conclusions

This study aimed to explore the complex regulatory networks in *S. clavuligerus*, with a focus on the role of sRNAs in gene expression and the biosynthetic pathways of secondary metabolites. By combining an RNA-seq analysis with bioinformatic predictions, we gained significant insights into the organism’s transcriptional landscape. As a result, the following conclusions can be drawn from the study:

*sRNA Enrichment in Conserved IGRs*: The discovery that conserved IGRs in *S. clavuligerus* are disproportionately enriched for small RNAs (sRNAs) is a major breakthrough. With a ratio of 19 known sRNAs identified out of 606 conserved IGRs, nearly five times higher than the occurrence in non-conserved IGRs, this highlights the evolutionary significance of these regions in sRNA regulation. This finding emphasizes that conserved IGRs are crucial reservoirs of regulatory elements, which could play pivotal roles in gene expression control across different environmental conditions.

*Genome-Wide Expression of Biosynthetic Gene Clusters*: The fact that the entire genome, including all BGCs, is functionally active is a significant revelation. This broad expression pattern suggests that *S. clavuligerus* possesses the potential to produce a vast array of secondary metabolites, many of which remain uncharacterized. This opens new opportunities to investigate less-explored BGCs and correlate their expression patterns with specific culture conditions, potentially leading to the discovery of novel bioactive compounds.

*Impact of sRNAs on Metabolism*: This study confirms the extensive role that sRNAs play in regulating key metabolic processes in *S. clavuligerus*, particularly in secondary metabolite production and amino acid biosynthesis. The identification of these sRNAs underscores the intricate layer of post-transcriptional regulation involved in metabolic pathways. Future studies that expand on these findings, including the testing of additional conditions and validation through mRNA sequencing, will provide deeper insights into the regulatory networks governed by sRNAs.

## Figures and Tables

**Table 1:** A summary of the number of transcripts assembled by Rockhopper using multiple RNA-seq datasets.

	Chromosome	Plasmid	Total
transcripts	11,456	2904	14,360
annotated	5546	1451	6997
predicted RNA	5910	1453	7363
antisense	4005	908	4913
sRNA	1905	545	2450

## Data Availability

The data presented in this study are available in the Sequence Read Archive (SRA) database in NCBI (Accession No. PRJNA1125019).

## Supplementary Materials

The following supporting information can be downloaded at https://www.mdpi.com/article/10.3390/ijms252413472/s1.
