# Supplementary material for: Identification of Small RNAs in Streptomyces clavuligerus Using High-Resolution Transcriptomics and Expression Profiling During Clavulanic Acid Production
Source: Int J Mol Sci. 2024 Dec 16;25(24):13472. doi: 10.3390/ijms252413472 (PMC11678152; doi:10.3390/ijms252413472)
Supplement: Supplementary file 1 [file ijms-25-13472-s001.zip › Supplementary_Figures_Tables_ijms-3303072.pdf]

Supplementary material

Identification of Small RNAs in *Streptomyces clavuligerus* Using High-Resolution Transcriptomics and Expression Profiling During Clavulanic Acid Production

Carlos Caicedo-Montoya [1], Luisa F. Patiño [1] and Rigoberto Ríos-Estepa [2,*]

[1] Grupo de Bioprocesos, Departamento de Ingeniería Química, Universidad de Antioquia UdeA,
Calle 70 No. 52-21, Medellín 050010, Colombia; candres.caicedo@udea.edu.co (C.C.-M.);
luisa.patinoc@udea.edu.co (L.F.P.)

[2] Grupo de Investigación en Simulación, Diseño, Control y Optimización de Procesos (SIDCOP),
Departamento de Ingeniería Química, Universidad de Antioquia, Medellín 050010, Colombia

* Correspondence: rigoberto.rios@udea.edu.co; Tel.: +57-4-2198568

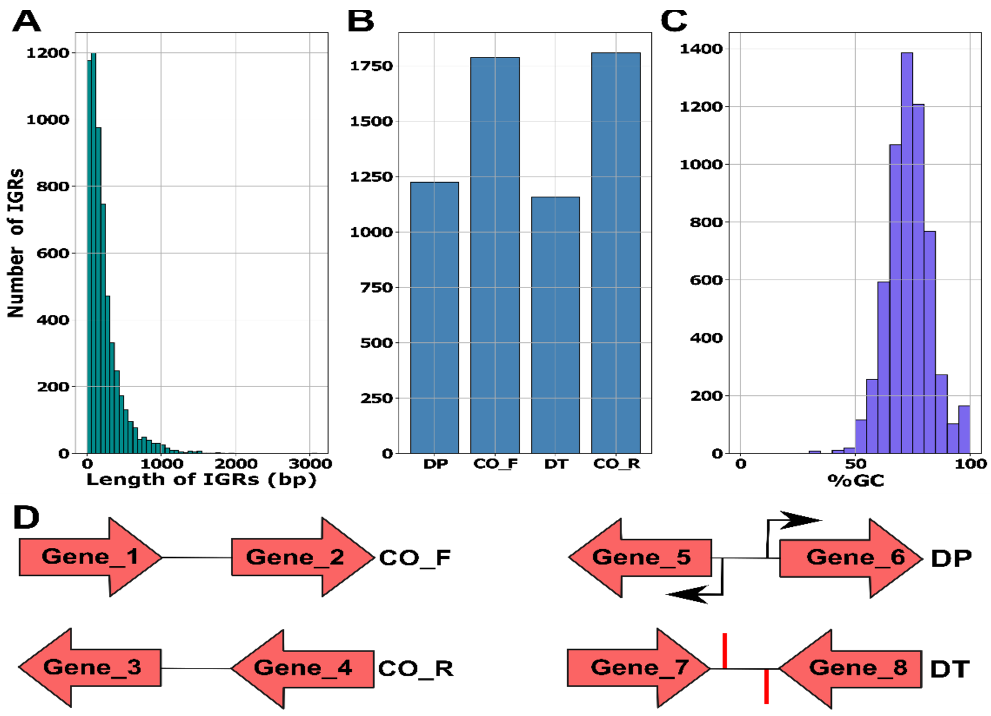

Supplementary Figure S1. General features of IGRs in *Streptomyces clavuligerus*. (**A**) Distribution of IGR lengths. (**B**) Classification of IGRs. (**C**) GC content of the IGRs. (**D**) Classification of IGRs according to their neighboring genes; genes are depicted as thick arrows. Double Promoter (DP) flanking genes oriented in divergent direction, Double Terminator (DT) flanking genes oriented in convergent direction, Co-oriented Forward (CO_F) flanking genes oriented in the same directions in forward strand, and Co-oriented Reverse (CO_R) flanking genes oriented in

the same directions in reverse strand. Presence of a promoter and a terminator in a flanking gene are indicated by an elbow arrow and a red line, respectively.

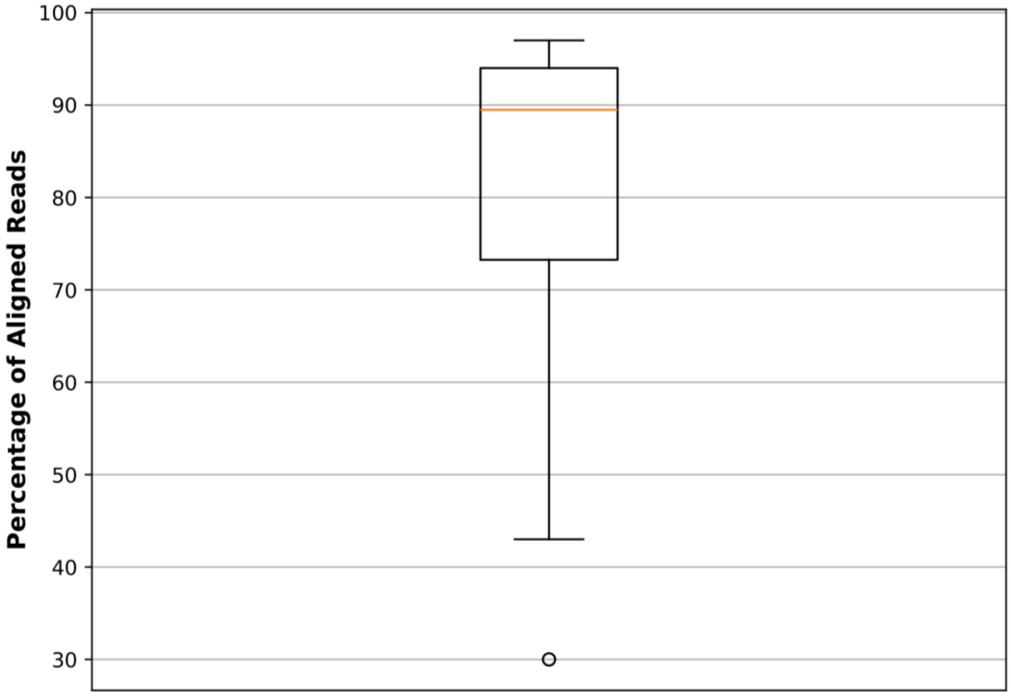

Supplementary Figure S2. Boxplot showing the percentage of reads aligned to the genome of *S. clavuligerus* ATCC 27064 from multiple RNA-seq samples, both downloaded from the SRA database and generated in this study.

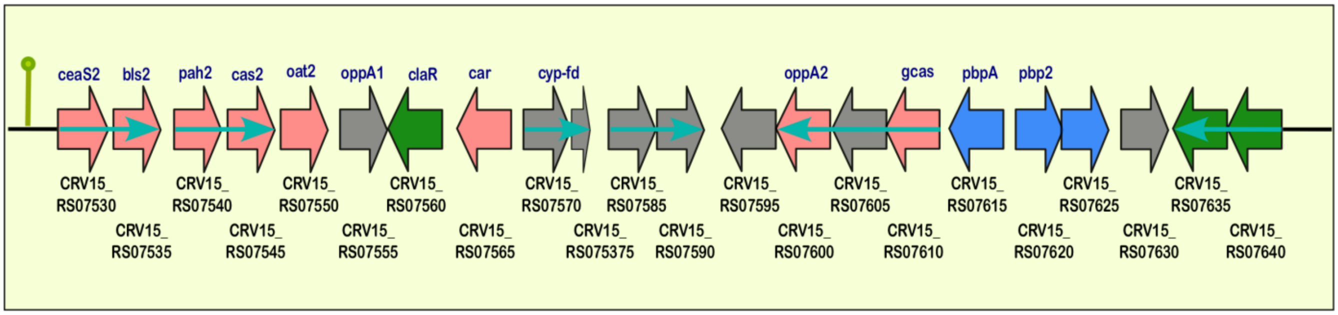

Supplementary Figure S3. Operon structure of the clavulanic acid biosynthetic gene cluster as determined from RNA-seq data using Rockhopper. Genes are represented by thick horizontal arrows. Transcriptional units (TUs) are depicted as groups of genes bound together by a light blue arrow. Green: transcriptional regulator; pale red: biosynthetic genes; grey: other genes; blue: transport-related genes; vertical green line: DasR binding site, a repressor dependent on N-acetylglucosamine.

**Identifier**	**Product**	**Length (nt)**	**Length (aa)**	**Function**
CRV15_07530	asparagine synthase	1542	513	biosynthetic
CRV15_07535	agmatinase	942	313	other
CRV15_07540	clavaminate synthase	978	325	biosynthetic
CRV15_07545	ornithine acetyltransferase	1182	393	biosynthetic-additional
CRV15_07550	ABC transporter substrate-binding protein	1668	555	transport
CRV15_07555	transcriptional regulator	1299	432	regulatory
CRV15_07560	KR domain-containing protein	747	248	biosynthetic-additional
CRV15_07565	cytochrome P450	1227	408	biosynthetic-additional
CRV15_07570	ferredoxin	207	68	other
CRV15_07575	glutathione peroxidase	87	29	other
CRV15_07580	clavulanate biosynthesis protein 12	1377	458	other
CRV15_07585	EamA family transporter	1023	340	transport
CRV15_07590	N-acetyltransferase	1020	339	other

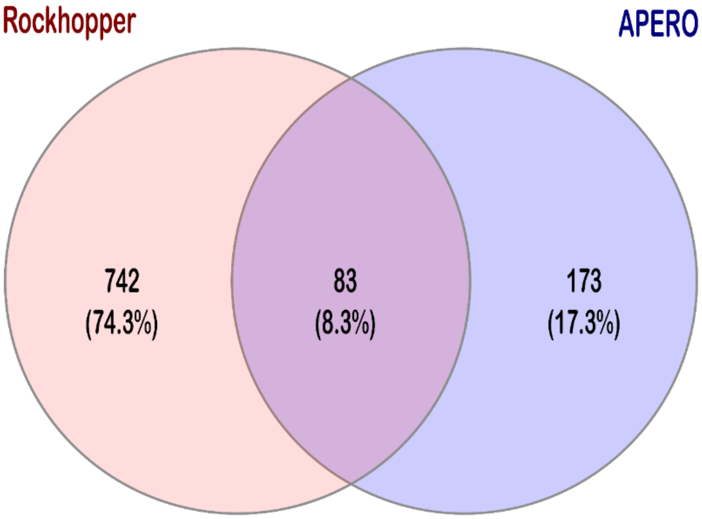

Supplementary Figure S4. Number of predicted sRNAs by Rockhopper and APERO.

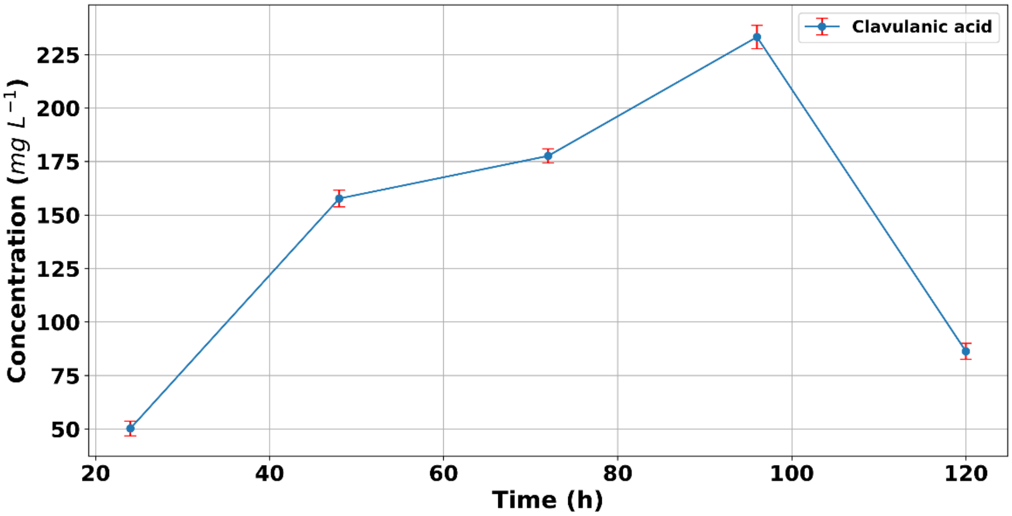

Supplementary Figure S5. Clavulanic acid production in soy protein isolate (ISP) medium.

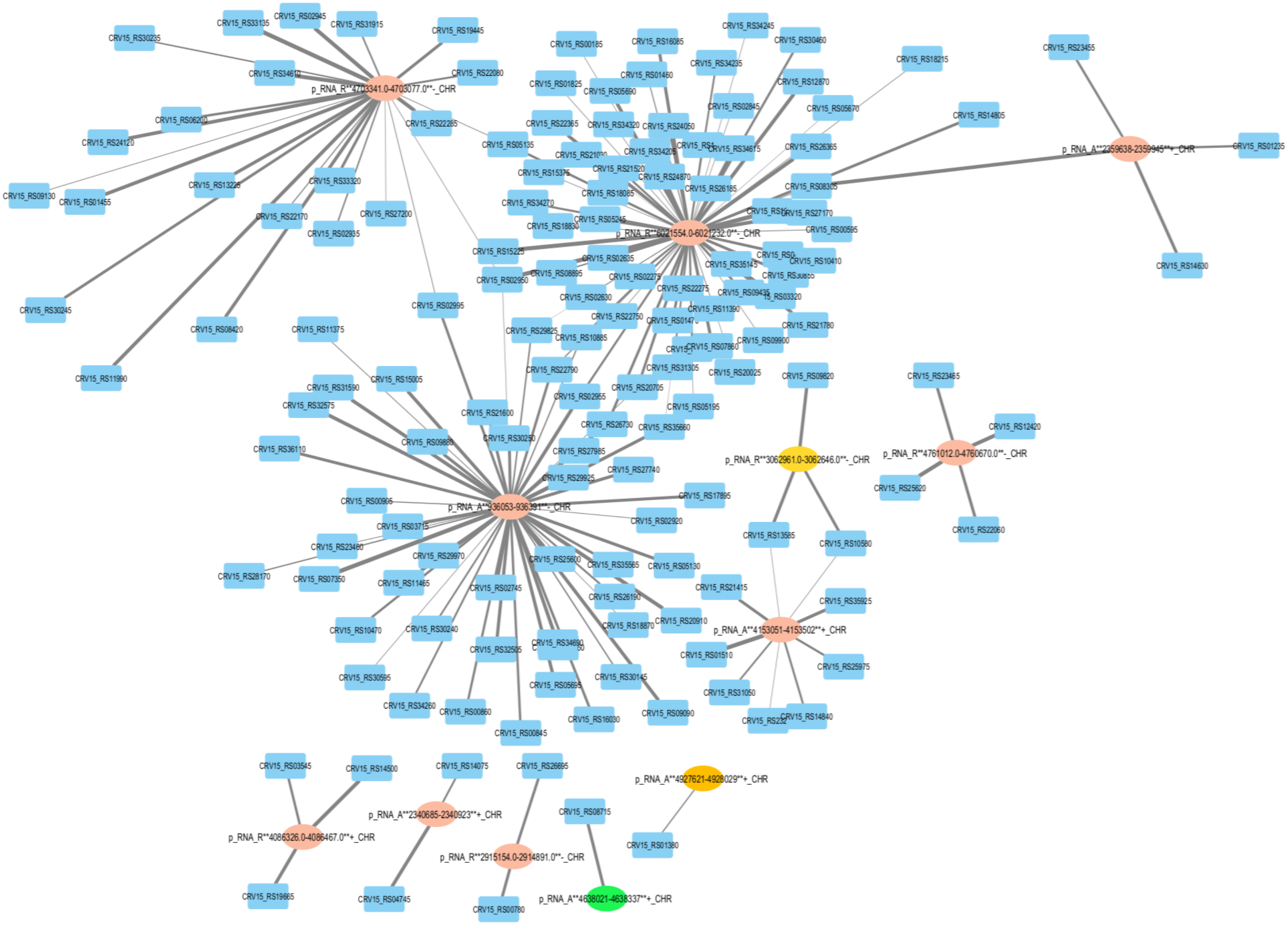

Supplementary Figure S6. RNA-mRNA interactions predicted by IntaRNA 2.0 and TargetRNA3 for the differentially expressed (DE) sRNAs at 96 hours. This interaction network shows downregulated sRNAs. Blue circles represent mRNA genes. Yellow circles indicate sRNAs from *S. clavuligerus* annotated in RFAM (if annotated); green circles indicate sRNAs annotated in other organisms, and red circles indicate unknown sRNAs. Thicker edges represent stronger interactions. Networks were generated using Cytoscape.

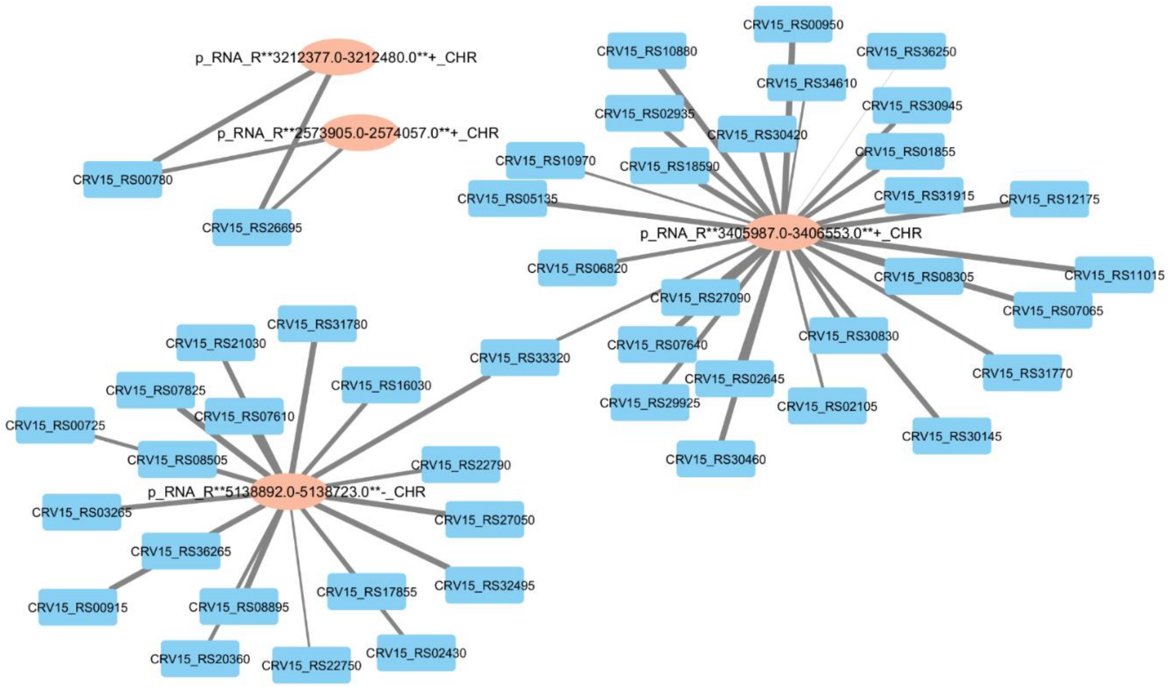

Supplementary Figure S7. RNA-mRNA interactions predicted by IntaRNA 2.0 and TargetRNA3 for the differentially expressed (DE) sRNAs at 96 hours. This interaction network shows up-regulated sRNAs. Blue circles represent mRNA genes. Yellow circles indicate sRNAs from *S. clavuligerus* annotated in RFAM (if annotated); green circles indicate sRNAs annotated in other organisms, and red circles indicate unknown sRNAs. Thicker edges represent stronger interactions. Networks were generated using Cytoscape.

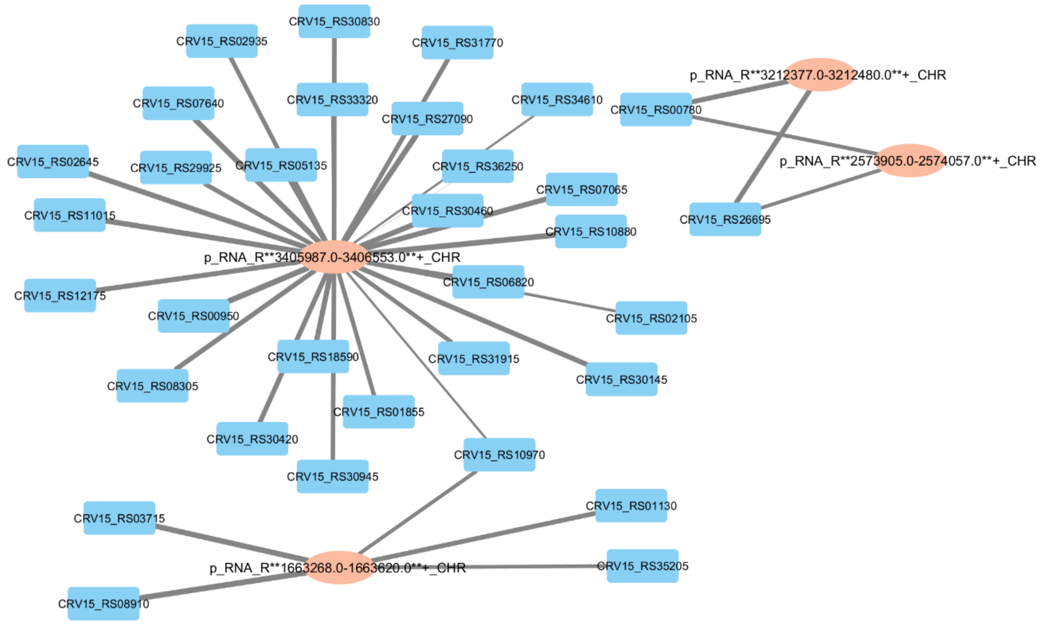

Supplementary Figure S8. RNA-mRNA interactions predicted by IntaRNA 2.0 and TargetRNA3 for the differentially expressed (DE) sRNAs at 72 hours. This interaction network shows upregulated sRNAs. Blue circles represent mRNA genes. Yellow circles indicate sRNAs from *S. clavuligerus* annotated in RFAM (if annotated); green circles indicate sRNAs annotated in other organisms, and red circles indicate unknown sRNAs. Thicker edges represent stronger interactions. Networks were generated using Cytoscape.

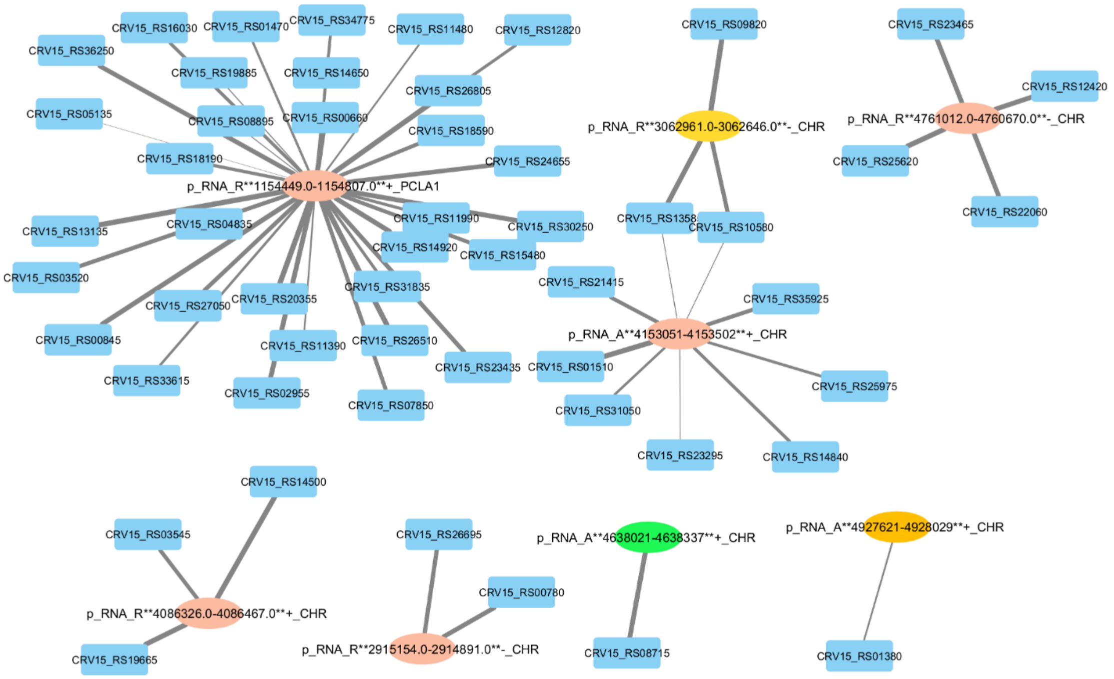

Supplementary Figure S9. RNA-mRNA interactions predicted by IntaRNA 2.0 and TargetRNA3 for the differentially expressed (DE) sRNAs at 72 hours. This interaction network shows down-regulated sRNAs. Blue circles represent mRNA genes. Yellow circles indicate sRNAs from *S. clavuligerus* annotated in RFAM (if annotated); green circles indicate sRNAs annotated in other organisms, and red circles indicate unknown sRNAs. Thicker edges represent stronger interactions. Networks were generated using Cytoscape.

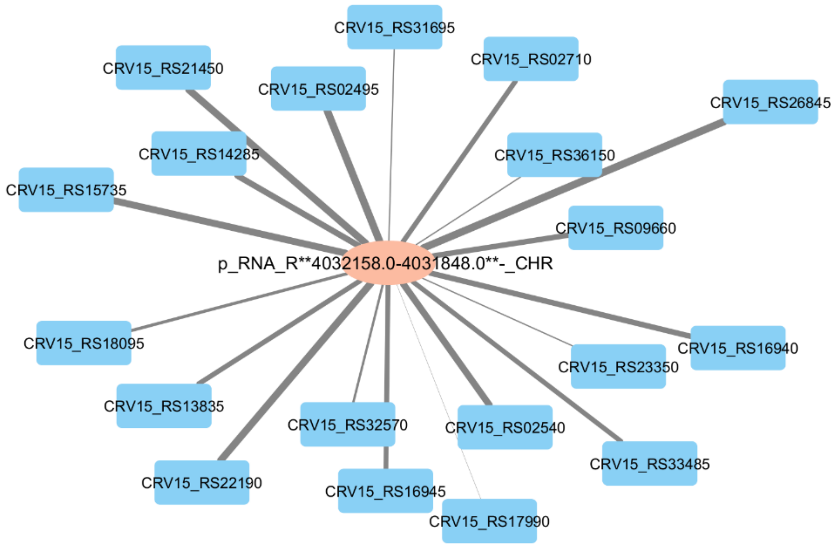

Supplementary Figure S10. RNA-mRNA interactions predicted by IntaRNA 2.0 and TargetRNA3 for the differentially expressed (DE) sRNAs at 48 hours. This interaction network shows upregulated sRNAs. Blue circles represent mRNA genes. Yellow circles indicate sRNAs from *S. clavuligerus* annotated in RFAM (if annotated); green circles indicate sRNAs annotated in other organisms, and red circles indicate unknown sRNAs. Thicker edges represent stronger interactions. Networks were generated using Cytoscape.

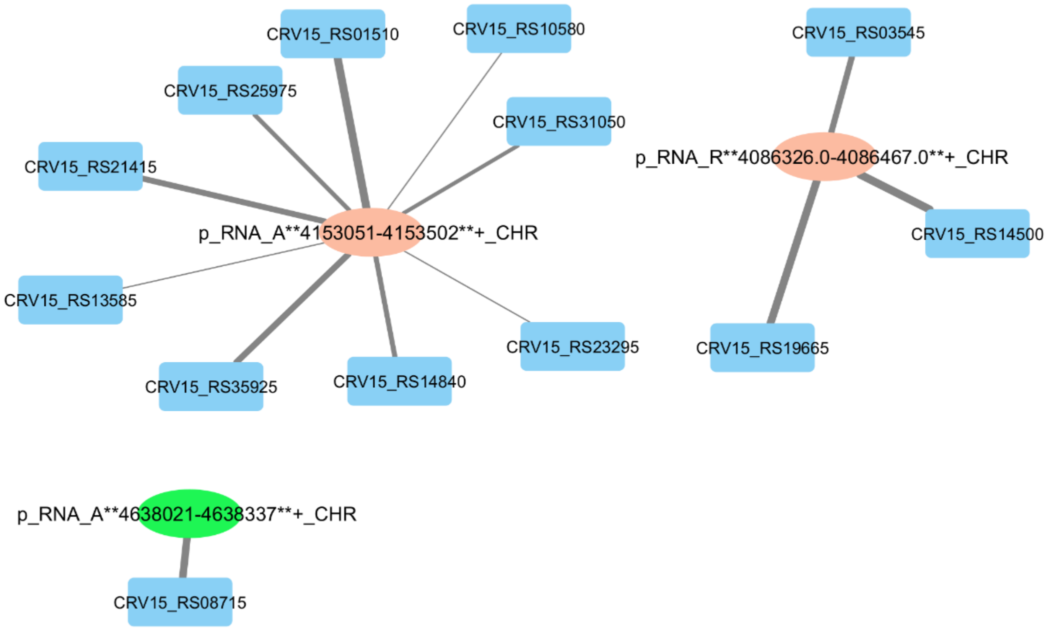

Supplementary Figure S11. RNA-mRNA interactions predicted by IntaRNA 2.0 and TargetRNA3 for the differentially expressed (DE) sRNAs at 48 hours. This interaction network shows down-regulated sRNAs. Blue circles represent mRNA genes. Yellow circles indicate sRNAs from *S. clavuligerus* annotated in RFAM (if annotated); green circles indicate sRNAs annotated in other organisms, and red circles indicate unknown sRNAs. Thicker edges represent stronger interactions. Networks were generated using Cytoscape.

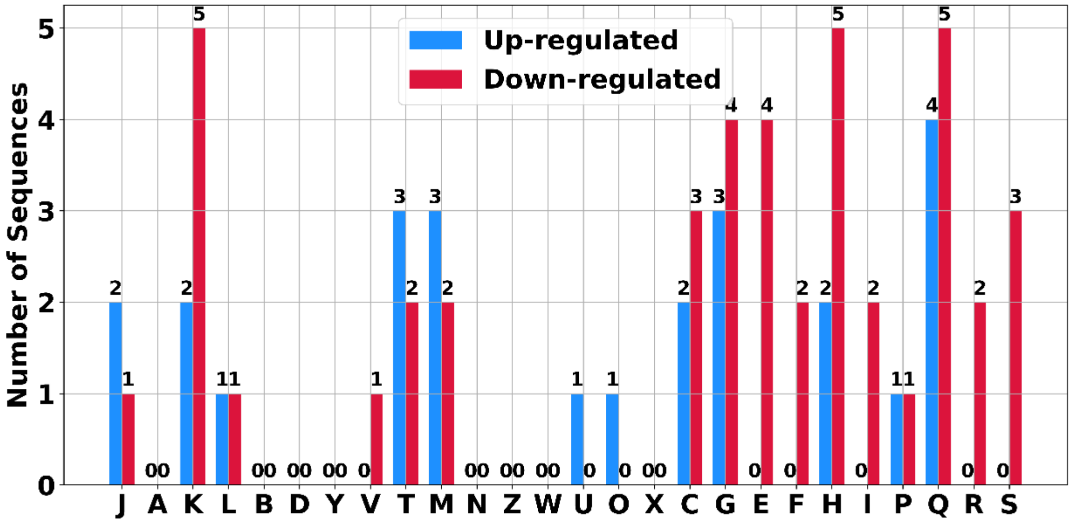

**J**	**Translation, ribosomal structure and biogenesis**	**U**	**Intracellular trafficking, secretion, and vesicular transport**
**A**	**RNA processing and modification**	**O**	**Posttranslational modification, protein turnover, chaperones**
**K**	**Transcription**	**X**	**Mobilome: prophages, transposons**
**L**	**Replication, recombination and repair**	**C**	**Energy production and conversion**
**B**	**Chromatin structure and dynamics**	**G**	**Carbohydrate transport and metabolism**
**D**	**Cell cycle control, cell division, chromosome partitioning**	**E**	**Amino acid transport and metabolism**
**Y**	**Nuclear structure**	**F**	**Nucleotide transport and metabolism**
**V**	**Defense mechanisms**	**H**	**Coenzyme transport and metabolism**
**T**	**Signal transduction mechanisms**	**I**	**Lipid transport and metabolism**
**M**	**Cell wall/membrane/envelope biogenesis**	**P**	**Inorganic ion transport and metabolism**
**N**	**Cell motility**	**Q**	**Secondary metabolites biosynthesis, transport and catabolism**
**Z**	**Cytoskeleton**	**R**	**General function prediction only**
**W**	**Extracellular structures**	**S**	**Function unknown**

Supplementary Figure S12. Cluster of Orthologous Groups (COG) functional classification for gene products from mRNAs potentially interacting with the differentially expressed (DE) sRNAs at 72 hours.

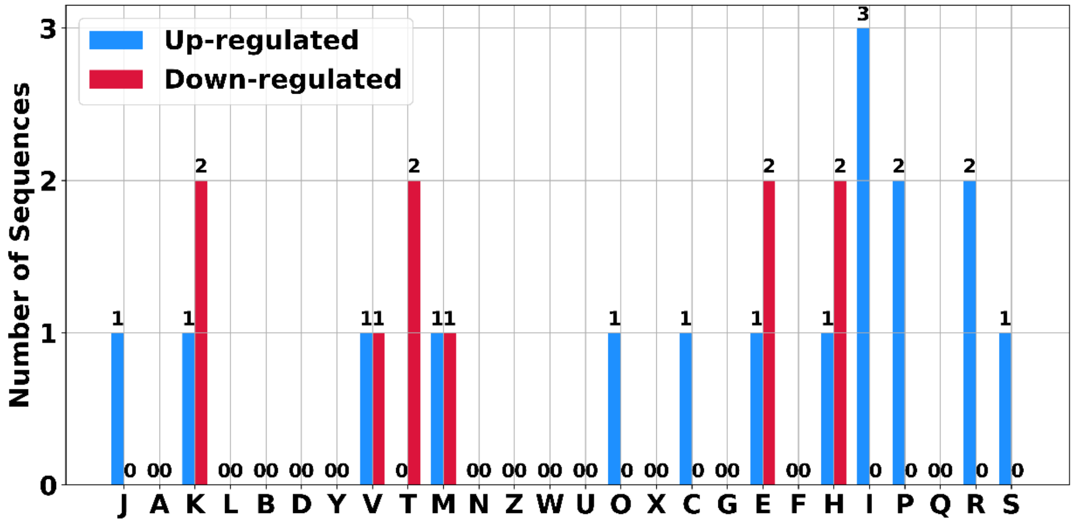

J	Translation, ribosomal structure and biogenesis	U	Intracellular trafficking, secretion, and vesicular transport
A	RNA processing and modification	O	Posttranslational modification, protein turnover, chaperones
K	Transcription	X	Mobilome: prophages, transposons
L	Replication, recombination and repair	C	Energy production and conversion
B	Chromatin structure and dynamics	G	Carbohydrate transport and metabolism
D	Cell cycle control, cell division, chromosome partitioning	E	Amino acid transport and metabolism
Y	Nuclear structure	F	Nucleotide transport and metabolism
V	Defense mechanisms	H	Coenzyme transport and metabolism
T	Signal transduction mechanisms	I	Lipid transport and metabolism
M	Cell wall/membrane/envelope biogenesis	P	Inorganic ion transport and metabolism
N	Cell motility	Q	Secondary metabolites biosynthesis, transport and catabolism
Z	Cytoskeleton	R	General function prediction only
W	Extracellular structures	S	Function unknown

Supplementary Figure S13. Cluster of Orthologous Groups (COG) functional classification for gene products from mRNAs potentially interacting with the differentially expressed (DE) sRNAs at 48 hours.

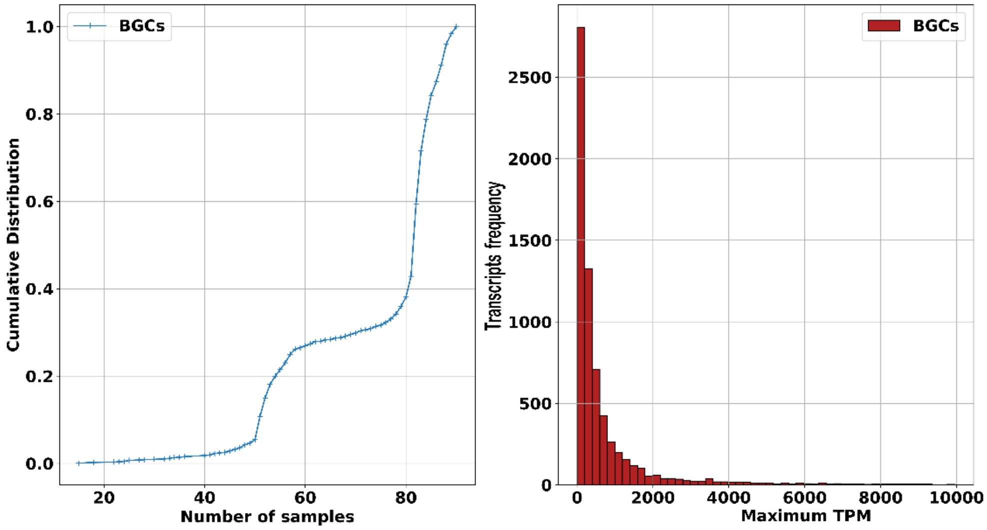

Supplementary Figure S14. Cumulative distribution of the fraction of transcripts expressed in a given number of RNA-seq samples (left); and frequency of transcripts expressed (right), determined by TPM, in each number of RNA-seq samples for all annotated genes belonging to a BGC.

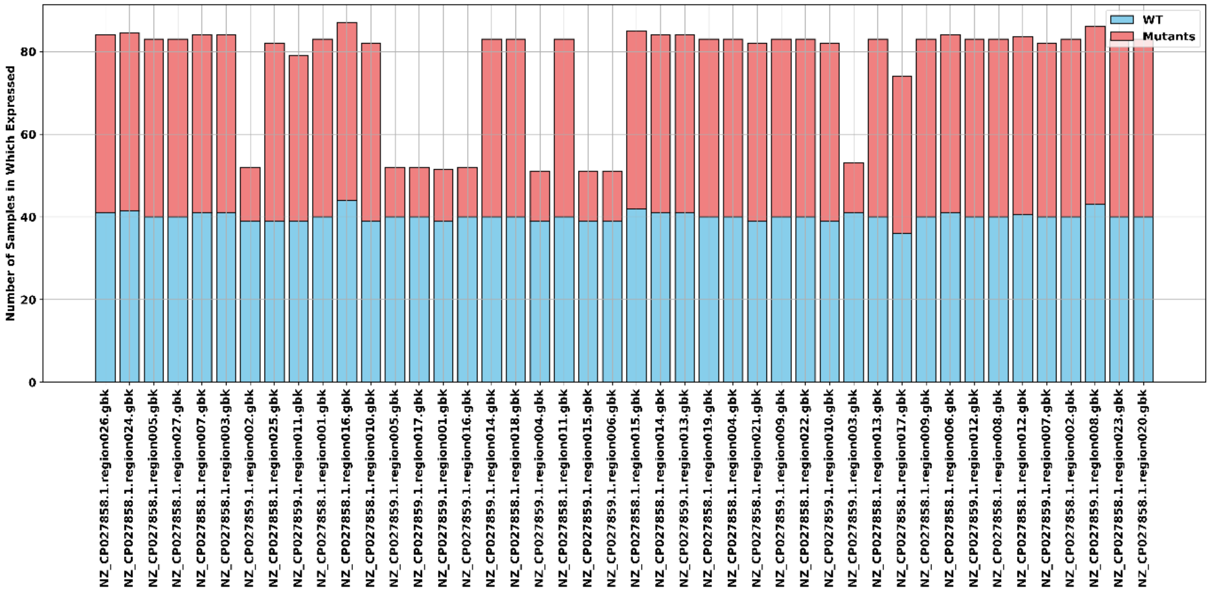

Supplementary Figure S15. Average number of samples in which the different regions harboring BGCs are expressed. WT: Wild-type strains. Description of each region is presented in Supplementary Table S3

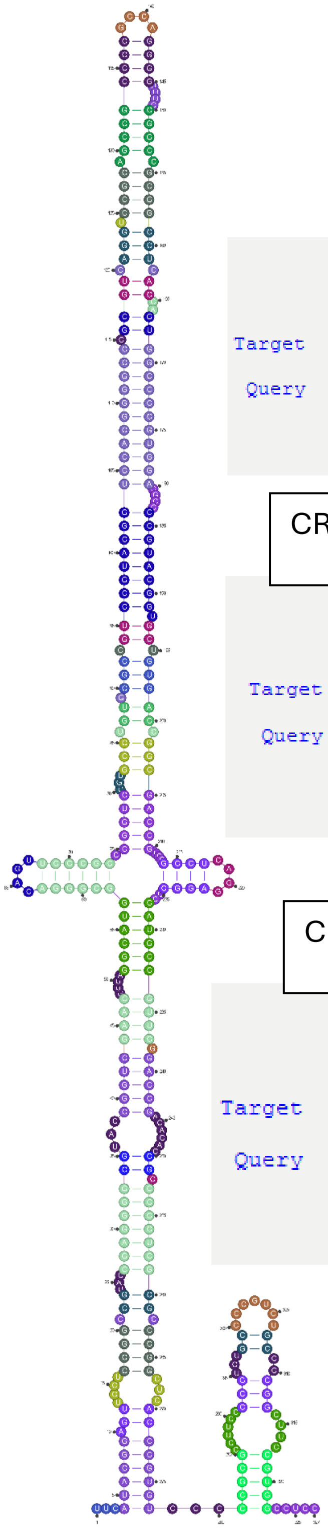

CRV15_RS19020(Energy -37.59 kcal/mol

```
                     5
                      |
               5'-AUGA  U          GA      GC          CGGACCC      C    AU      G  GC
Target                CG  CUGA ACAC  GCCCCC  CGCGUCCGGC       UCACCC   UC  CCGUGC CU  UC
                      ||  |||| ||||  ||||||  ||||||||||       ||||||   ||  |||||| ||  ||
 Query                GC  GGCU UGUG  CGGGGG  GCGCGGCCG        AGUGGG   AG  GGCGCG GA  AG
         3'-AUG..GGCAA  CU    G    G       A                        CAC  GG
                      |
                    90
```

CRV15_RS19020 (Energy -48.17 kcal/mol

```
                   702
                      |
         5'-AUG..GUCGC     AACA       GC        A     CUGC     AGCA        CU  CUCA    G
Target                CCCCG     GCUCCG     UGCUG GGCUG    GCGGC    CCGCC CA  GG    CCCC
                      |||||     ||||||     ||||| |||||    |||||    ||||| ||  ||    ||||
 Query                GGGGC     CGGGGC     ACGAC CUGGC    CGCUG    GGCGG GU  CC    GGGG
         3'-AUG..UCACC     ACCUG      AUGCC     A              GCAA     A      UCC     U
                      |
                   156
```

CRV15_RS15225 (Energy -33.41 kcal/mol

```
                   522                                          556
                      |                                         |
         5'-AUG..GGGCU          UU     UG              C        GGCUC..UGA-3'
Target                CGCCUUCCCC  UCUGC     CUGCCCUGGCU     GGCC
                      ||||||||||  |||||     |||||||||||     ||||
 Query                GCGGAAGGGG  AGACG     GACGGGGCCGA     CCGG
         3'-AUG..GGGGC          C      GGGCA           AGGCA    AG-5'
                      |                                         |
                    43                                          3
```

Supplementary Figure S16. Secondary structure of "p_RNA_R_6021554-6021232 (Left). The structure was constructed by RNArtist. Regions of interaction between the predicted sRNA and some selected targets (Right)

RNArtist is available online: https://github.com/fjossinet/RNArtist

Supplementary Table S1. NCBI accession numbers for genome assemblies used in the IGR comparison

Strain	Accession Number
Streptomyces globosus soil	GCF_003325375.1_ASM332537v1
Streptomyces lavendulae lavendulae CCM 3239	GCF_002803845.1_ASM280384v1
Streptomyces sp. Mg1	GCF_000412265.2_ASM41226v2
Streptomyces sp. fd1-xmd	GCF_002007685.1_ASM200768v1
Streptomyces sp. TN58	GCF_001941845.1_ASM194184v1
Streptomyces sp. Sge12	GCF_002080455.1_ASM208045v1
Streptomyces sp. 3211	GCF_002028385.1_ASM202838v1
Streptomyces spongiicola HNM0071	GCF_003122365.1_ASM312236v1
Streptomyces tirandamycinicus HNM0039	GCF_003097515.1_ASM309751v1
Streptomyces rubrolavendulae MJM4426	GCF_001750785.1_ASM175078v1
Streptomyces exfoliatus A1013Y	GCF_005517195.1_ASM551719v1
Streptomyces sp. WAC00288	GCF_002943895.1_ASM294389v1
Streptomyces venezuelae NRRL B-65442	GCF_001886595.1_ASM188659v1
Streptomyces vietnamensis GIMV4.0001	GCF_000830005.1_ASM83000v1
Streptomyces lunaelactis MM109	GCF_003054555.1_ASM305455v1
Streptomyces pristinaespiralis HCCB 10218	GCF_001278075.1_ASM127807v1
Streptomyces clavuligerus ATCC 27064	GCF_005519465.1_ASM551946v1
Streptomyces clavuligerus F1D-5	GCF_003454755.1_ASM345475v1
Streptomyces clavuligerus F613-1	GCF_001693675.1_ASM169367v1
Streptomyces sp. GSSD-12	GCF_003344965.1_ASM334496v1
Streptomyces niveus SCSIO 3406	GCF_002009175.1_ASM200917v1
Streptomyces atratus SCSIO ZH16	GCF_003330865.1_ASM333086v1
Streptomyces sp. SM18	GCF_002910775.2_ASM291077v2
Streptomyces sp. Sv. ACTE SirexAA-E	GCF_000177195.2_ASM17719v2
Streptomyces sp. PAMC26508	GCF_000364805.1_ASM36480v1
Streptomyces pratensis ATCC 33331	GCF_000176115.2_ASM17611v2
Streptomyces fulvissimus DSM 40593	GCF_000385945.1_ASM38594v1
Streptomyces sp. S8	GCF_002094995.1_ASM209499v1
Streptomyces sp. CFMR 7	GCF_001278095.1_ASM127809v1
Streptomyces bacillaris ATCC 15855	GCF_003268675.1_ASM326867v1
Streptomyces sp. DUT11	GCF_002848525.1_ASM284852v1
Streptomyces violaceoruber S21	GCF_002082175.1_ASM208217v1
Streptomyces griseus NBRC 13350	GCF_000010605.1_ASM1060v1
Streptomyces sp. S063	GCF_002832675.1_ASM283267v1
Streptomyces sp. Tue6075	GCF_001931635.1_ASM193163v1
Streptomyces globisporus C-1027	GCF_000261345.2_ASM26134v2
Streptomyces globisporus TFH56	GCF_003147545.1_ASM314754v1

Supplementary Table S2. Largest operons found in *S. clavuligerus* genome by Rockhopper.

Replicon	Start	Stop	Strand	Number of Genes	Genes	Function
**Chromosome**	2507976	2514801	-	15	CRV15_RS10220, rplE, rplX, rplN, rpsQ, rpmC, rplP, rpsC, rplV, rpsS, rplB, rplW, rplD, rplC, rpsJ	Ribosomal Proteins
**Chromosome**	650154	664570	-	14	CRV15_RS02615, CRV15_RS02620, CRV15_RS02625, CRV15_RS02630, CRV15_RS02635, CRV15_RS02640, CRV15_RS02645, CRV15_RS02650, CRV15_RS02655, CRV15_RS02660, CRV15_RS02665, CRV15_RS02670, CRV15_RS02675, CRV15_RS35805	Unknown
**Chromosome**	2584193	2600408	-	14	nuoN, CRV15_RS10615, nuoL, nuoK, CRV15_RS10630, nuoI, nuoH, CRV15_RS10645, nuoF, nuoE, CRV15_RS10660, CRV15_RS10665, CRV15_RS10670, CRV15_RS10675	NADH-quinone oxidoreductase
**Chromosome**	6640616	6653838	-	13	CRV15_RS27890, CRV15_RS27895, CRV15_RS27900, CRV15_RS27905, CRV15_RS27910, CRV15_RS27915, CRV15_RS27920, CRV15_RS27925, CRV15_RS27930, CRV15_RS27935, CRV15_RS27940,	Unknown

					CRV15_RS27945, CRV15_RS27950	
**Chromosome**	67910	79603	-	11	rfbC, CRV15_RS00305, CRV15_RS00310, CRV15_RS00315, CRV15_RS00320, CRV15_RS00325, CRV15_RS00330, CRV15_RS00335, CRV15_RS00340, CRV15_RS00345, CRV15_RS00350	10-epi-HSAF
**Chromosome**	3812903	3821924	+	11	CRV15_RS15945, CRV15_RS15950, CRV15_RS15955, CRV15_RS15960, CRV15_RS35950, CRV15_RS15965, CRV15_RS15970, CRV15_RS15975, CRV15_RS15980, CRV15_RS15985, CRV15_RS15990	Unknown
**Chromosome**	1794835	1803517	+	10	CRV15_RS07120, CRV15_RS07125, CRV15_RS07130, galE, CRV15_RS07140, CRV15_RS07145, CRV15_RS07150, CRV15_RS07155, CRV15_RS07160, CRV15_RS07165	Tunicamycin B1 biosynthetic gene cluster
**Chromosome**	2548305	2558682	-	10	CRV15_RS10460, CRV15_RS10465, CRV15_RS10470, nuoK, CRV15_RS10480, CRV15_RS10485, CRV15_RS10490, CRV15_RS10495,	NADH-quinone oxidoreductase

					CRV15_RS10500, CRV15_RS10505	
**Plasmid**	286029	305141	-	12	CRV15_RS29555, CRV15_RS29560, CRV15_RS29565, CRV15_RS29570, sbnB, sbnA, CRV15_RS29585, CRV15_RS29590, CRV15_RS29595, CRV15_RS29600, CRV15_RS29605, CRV15_RS29610	(-)-δ-cadinene biosynthetic gene cluster
**Plasmid**	1033163	1044045	+	11	CRV15_RS32770, pgl, CRV15_RS32780, CRV15_RS32785, CRV15_RS32790, CRV15_RS32795, CRV15_RS32800, CRV15_RS32805, CRV15_RS32810, CRV15_RS32815, CRV15_RS32820	Low similarity to depsibosamycin B biosynthetic gene cluster
**Plasmid**	426676	433468	+	8	CRV15_RS30215, CRV15_RS30220, CRV15_RS30225, CRV15_RS30230, CRV15_RS30235, CRV15_RS30240, CRV15_RS30245, CRV15_RS30250	Unknown

Supplementary Table S3. Antismash Predictions for *S. clavuligerus*

Region	Type	From	To	Most similar known cluster		Similarity
**Region 1.1**	NI-siderophore	2,794	34,362	peucechelin	NRP	25%
**Region 1.2**	NRPS-like,other,NRPS,T1PKS,terpene	77,561	175,333	SGR PTMs/SGR PTM Compound b/SGR PTM Compound c/SGR PTM Compound d	NRP+Polyketide	83%
**Region 1.3**	T3PKS,ranthipeptide	278,060	319,115	naringenin	Polyketide:Type III polyketide	100%
**Region 1.4**	NRPS,NRPS-like	459,596	508,869	nucleocidin	Other	47%
**Region 1.5**	NRPS	527,573	570,863	holomycin	NRP	100%
**Region 1.6**	terpene	578,227	605,009	hopene	Terpene	69%
**Region 1.7**	NRPS	684,211	727,552			
**Region 1.8**	redox-cofactor	750,603	772,973	lankacidin C	NRP+Polyketide	20%
**Region 1.9**	lanthipeptide-class-iii,T2PKS	926,046	1,019,570	spore pigment	Polyketide	83%
**Region 1.10**	RiPP-like	1,041,263	1,052,636			
**Region 1.11**	NRPS	1,163,792	1,242,550	A-201A	Other	15%
**Region 1.12**	NI-siderophore	1,282,132	1,312,183	kinamycin	Polyketide	19%
**Region 1.13**	T1PKS,NRPS	1,543,626	1,598,790	asukamycin	Polyketide:Type II polyketide	3%
**Region 1.14**	lanthipeptide-class-i	1,653,875	1,679,161			
**Region 1.15**	nucleoside	1,782,683	1,803,654	tunicamycin B1	Other:Nucleoside	85%
**Region 1.16**	NRPS,blactam	1,861,345	1,912,633	cephamycin C	NRP:Beta-lactam	84%
**Region 1.17**	melanin	2,231,583	2,242,017	melanin	Other	100%

**Region 1.18**	blactam	3,301,354	3,322,328	alanylclavam/2-hydroxymethylclavam/2-formyloxymethylclavam/clavam-2-carboxylate	Other:Non-NRP beta-lactam	75%
**Region 1.19**	aminopolycarboxylic-acid	3,561,811	3,575,400	EDHA	Other	77%
**Region 1.20**	lanthipeptide-class-i	3,834,469	3,859,566			
**Region 1.21**	butyrolactone	4,011,689	4,022,657	lactonamycin	Polyketide	3%
**Region 1.22**	NRPS	4,028,923	4,076,251	clipibicyclene/azabicyclene B/azabicyclene C/azabicyclene D	NRP+Polyketide	11%
**Region 1.23**	NI-siderophore	4,422,537	4,452,357	desferrioxamin B	Other	100%
**Region 1.24**	ectoine	5,423,823	5,434,248	ectoine	Other	100%
**Region 1.25**	PKS-like,LAP,butyrolactone,T1PKS	6,066,295	6,153,436	4-hexadecanoyl-3-hydroxy-2-(hydroxymethyl)-2H-furan-5-one	Polyketide	63%
**Region 1.26**	terpene	6,504,864	6,527,038	geosmin	Terpene	100%
**Region 1.27**	T1PKS	6,653,964	6,748,591	JBIR-100	Polyketide:Modular type I polyketide	72%
**Region 2.1**	terpene	76,246	97,502	(+)-T-muurolol	Terpene	80%
**Region 2.2**	NRPS,NRPS-like,terpene	255,612	334,765	(-)-δ-cadinene	Terpene	100%
**Region 2.3**	lassopeptide	390,856	413,322			
**Region 2.4**	terpene	471,472	492,503	cyslabdan	Terpene	18%

**Region 2.5**	indole,NRPS-like,terpene	538,336	599,445	abyssomicin M/abyssomicin N/abyssomicin O/abyssomicin P/abyssomicin Q/abyssomicin R/abyssomicin S/abyssomicin T/abyssomicin U/abyssomicin V/abyssomicin W/abyssomicin X	Polyketide	9%
**Region 2.6**	terpene,lanthipeptide-class-iv	643,291	670,905	venezuelin	RiPP:Lanthipeptide	100%
**Region 2.7**	terpene	800,510	821,475			
**Region 2.8**	butyrolactone	861,601	872,533			
**Region 2.9**	amglyccycl,terpene,T1PKS,NRPS	1,026,084	1,098,543	depsibosamycin B/depsibosamycin C/depsibosamycin D	NRP	23%
**Region 2.10**	blactam	1,142,847	1,164,448	alanylclavam/2-hydroxymethylclavam/2-formyloxymethylclavam/clavam-2-carboxylate/clavulanic acid	Other:Non-NRP beta-lactam	100%
**Region 2.11**	indole	1,199,815	1,223,432	staurosporine	Alkaloid	82%
**Region 2.12**	RiPP-like	1,229,724	1,241,718			
**Region 2.13**	terpene	1,286,162	1,318,471	primycin	Polyketide	5%
**Region 2.14**	melanin,T1PKS	1,334,215	1,388,170	neocarzinostatin	Polyketide:Iterative type I polyketide+Polyketide:Enediyne type I polyketide	17%

**Region 2.15**	T1PKS,NRPS-like,phosphoglycolipid,NRPS,ectoine	1,392,244	1,557,780	maduropeptin	Polyketide:Iterative type I polyketide+Polyketide:Enediyne type I polyketide	37%
**Region 2.16**	terpene,NRPS	1,594,503	1,656,265	rapamycin	NRP+Polyketide	7%
**Region 2.17**	NRPS-like	1,668,918	1,712,991	minimycin	NRP+Saccharide	40%
